# An Evaluation of the Safety, Immunogenicity, and Protective Efficacy of a Combined Diphtheria–Tetanus–Acellular Pertussis, *Haemophilus influenzae* Type b, and ACYW135 Meningococcal Conjugate Vaccine in Murine and Rat Models

**DOI:** 10.3390/vaccines13070724

**Published:** 2025-07-03

**Authors:** Xiuwen Sui, Zhujun Shao, Yuanyuan Ji, Hairui Wang, Qingfu Xu, Bochao Wei, Zhuojun Duan, Chang Wang, Ying Yang, Jiayu Zhao, Tao Zhu

**Affiliations:** 1 CanSino Biologics Inc., Tianjin 300457, China; xiuwen.sui@cansinotech.com (X.S.); yuanyuan.ji@cansinotech.com (Y.J.); qingfu.xu@cansinotech.com (Q.X.); bochao.wei@cansinotech.com (B.W.); zhuojun.duan@cansinotech.com (Z.D.); chang.wang@cansinotech.com (C.W.); ying.yang@cansinotech.com (Y.Y.); jiayu.zhao@cansinotech.com (J.Z.); 2 National Key Laboratory of Intelligent Tracking and Forecasting for Infectious Diseases, National Institute for Communicable Disease Control and Prevention, Chinese Center for Disease Control and Prevention, Beijing 102206, China; shaozhujun@icdc.cn (Z.S.); wanghairui@icdc.cn (H.W.)

**Keywords:** Combined Diphtheria-Tetanus-Acellular Pertussis, *Haemophilus influenzae* Type b, ACYW135 Meningococcal Conjugate Vaccine, safety, immunogenicity, protective efficacy

## Abstract

**Background**: The combined diphtheria–tetanus–acellular pertussis (three-component), *Haemophilus influenzae* type b (Hib, conjugate), and ACYW135 meningococcal (conjugate) vaccine (DTaP-Hib-MCV4) offers a promising alternative to single-component vaccines, potentially simplifying immunization schedules and improving vaccination coverage. **Methods**: We evaluated the safety, immunogenicity, and protective efficacy of DTaP-Hib-MCV4 in animal models. Acute and long-term toxicity studies were conducted in Sprague-Dawley (SD) rats with equal numbers of male and female animals. Immunogenicity was assessed in female NIH mice and SD rats using a three-dose regimen at 14-day intervals. Orbital blood was collected 14 days post-immunization to measure IgG titers against pertussis, diphtheria, tetanus, Hib, and meningococcal antigens. The protective efficacy was determined using potency tests for the pertussis, diphtheria, and tetanus components; passive protection studies for Hib; and serum bactericidal antibody (SBA) titers against A/C/Y/W135 meningococcal serogroups. **Results**: Acute and repeated-dose toxicity studies in SD rats showed no signs of abnormal toxicity or irritation at either high (three doses/rat) or low (one dose/rat) doses levels. The no-observed-adverse-effect level (NOAEL) for DTaP-Hib-MCV4 was established at three doses/rat after 8 weeks of repeated intramuscular administration and a 4-week recovery period. Specific IgG antibodies against all the vaccine components were detected in animal sera at both one and three doses/rat, with no evidence of immunotoxicity. Following two-dose primary immunization in murine models, the combined vaccine elicited robust antigen-specific antibody responses, with geometric mean titers (GMTs) as follows: 1,280,000 for pertussis toxin (PT); 761,093 for filamentous hemagglutinin (FHA); 1,159,326 for pertactin (PRN); 1,659,955 for diphtheria toxoid (DT); 1,522,185 for tetanus toxoid (TT); 99 for *Haemophilus influenzae* type b (Hib); and 25,600, 33,199, 8300, and 9051 for serogroups A, C, Y, and W135 of *Neisseria meningitidis*, respectively. In the rat models, three-dose primary immunization also elicited robust antigen-specific antibody responses. Protection studies demonstrated efficacy against pertussis, tetanus toxin, and diphtheria toxin challenges. In the Hib challenge study, none of the 10 animals given anti-DTaP-Hib-MCV4 antiserum developed bacteremia after the live Hib challenge (vs. 5814/0.1 mL in the negative control, *p* < 0.001). In addition, the SBA titers against meningococcal serogroups exceeded the protective threshold (≥1:8) in 92.2% of the immunized mice and 100% of the immunized rats. Crucially, the combined vaccine induced potent immune responses and protective efficacy, with antibody levels and protection against each component antigen comparable to or greater than those of the individual components: DTaP, Hib, and MCV4. **Conclusions**: These findings demonstrate that the DTaP-Hib-MCV4 combined vaccine is both safe and immunogenic, supporting its potential as a viable alternative to individual vaccines. This combined vaccine may streamline immunization programs and enhance vaccination coverage.

## 1. Introduction

Infectious diseases, such as pertussis, diphtheria, tetanus, *Haemophilus influenzae* type b (Hib), and meningococcal infections, remain significant threats to global child health. Vaccination is widely recognized as the most effective strategy to prevent these diseases, having dramatically reduced their morbidity and mortality worldwide [1]. However, the increasing complexity of immunization schedules, driven by the introduction of new vaccines, has created substantial challenges. For instance, under many national immunization programs, children may receive more than 30 vaccine doses by the age of three [2]. This escalation in the number of required injections has led to logistical burdens, increased healthcare costs, and potential conflicts in vaccination timing, all of which can compromise immunization coverage [3]. In response, the development of combined vaccines has emerged as a transformative solution, offering the potential to reduce the number of injections, simplify immunization schedules, and improve overall vaccination rates [4]. The World Health Organization (WHO) has strongly advocated for the use of combined vaccines, emphasizing their ability to minimize dosing frequency, enhance coverage, and reduce the risk of adverse events [5]

The development of combination vaccines in China is undergoing transformative growth, driven by synergistic advancements in biotechnology and progressive immunization policy reforms. Internationally well-established pentavalent vaccines, exemplified by Sanofi’s DTaP-IPV/Hib formulation (Pentaxim^®^), have dominated the market for over a decade, providing comprehensive protection against diphtheria, tetanus, acellular pertussis, poliomyelitis, and *Haemophilus influenzae* type b (Hib) [6]. In China, infants under 1 year of age are at the highest risk of meningococcal meningitis. From 2015 to 2017, the annual incidence rate in this age group was 0.13 per 100,000, three-to-four times higher than other age groups. Since 2006, when the national surveillance system was set up, 721 infant cases (11% of all such cases) were reported by 2023. About 50% of these infant cases were in those under 6 months of age. For 0–5-month-old infants, the distribution was as follows: 14% (0–1 month), 14% (1–2 months), 16% (2–3 months), 21% (3–4 months), 17% (4–5 months), 18% (5–6 months). Though meningococcal vaccination has reduced China’s disease burden, an alarming change happened after 2015. The proportion of cases among 0–2-month-old infants within the <6-month-old group rose from 38% (before 2015) to 59% (after 2015) [7]. This trend was further confirmed by clinical reports, such as a serogroup Y meningococcal infection in a 5-month-old infant in Shantou City in 2019 [8] and a neonatal case (28 days old) with serogroup Y infection in Maoming City in 2020 [9]. Currently, the immunization protocols in China suggest initiating meningococcal polysaccharide conjugate vaccines at 3 months of age. However, this vaccination schedule leaves infants aged 0–2 months exposed and vulnerable during the pre-vaccination period, highlighting the urgent necessity for strengthened preventive strategies to target this crucial time window. From a vaccinology point of view, the strategic integration of DTP-based core antigens with supplemental components, such as Hib and meningococcal serogroups (ACYW135), makes a paradigm shift in combination vaccine development. Such multivalent formulations not only provide broad-spectrum protection against multiple pathogens but may also harness potential synergistic effects to enhance immune responses [10]. Recent studies have demonstrated both the feasibility and immunological benefits of these combined vaccines, showing that they can elicit robust and durable immunity while maintaining an acceptable safety profile [11]. The rationale for developing a combined vaccine incorporating diphtheria, tetanus, acellular pertussis (DTaP), Hib, and the ACYW135 meningococcal conjugate is particularly compelling, given the overlapping target populations and immunization schedules for these diseases. Infants and young children, who are the primary recipients of DTaP vaccines, are also highly susceptible to Hib and meningococcal infections [12]. By integrating these antigens into a single formulation, a combined vaccine could streamline immunization practice, reduce the number of required injections, and improve adherence to vaccination schedules, thereby addressing critical public health needs [13].

Preclinical studies are essential for evaluating the safety [14], immunogenicity, and protective efficacy of combined vaccines [15]. These studies provide critical insights into potential antigen–antigen interactions, formulation stability, and overall safety profiles, all of which are essential for guiding clinical development. Recent advancements in vaccine technology, including novel antigen conjugation techniques, innovative adjuvant systems [16], and optimized formulation designs, have further enhanced the feasibility of developing multivalent vaccines that are both immunogenic and stable. In this context, we have developed a novel combined vaccine comprising DTaP, Hib, and ACYW135 meningococcal conjugate components. This study aims to comprehensively evaluate the preclinical safety, immunogenicity, and protective efficacy of this vaccine in animal models, thereby establishing a foundation for future clinical trials.

For safety assessment, Sprague-Dawley (SD) rats were selected as the primary animal model and subjected to both rigorous acute and long-term toxicity studies. Immunogenicity was evaluated in both SD rats and NIH mice, two models with a well-documented utility in vaccine research. Specifically, SD rats were employed based on evidence that the Hib vaccine elicits immune responses in adult rats comparable to those observed in infants, making them a suitable model for assessing Hib vaccine formulations [17]. NIH mice, a standard model for evaluating DTaP and meningococcal vaccines, were used to ensure robust immunogenicity data across all the vaccine components [18,19]. The protective efficacy was assessed using a multifaceted approach: pharmacopoeia methods; passive immunization studies were conducted to evaluate protection against Hib [20]; and serum bactericidal antibody (SBA) assays were performed to determine the in vitro bactericidal activity of the meningococcal components [21,22].

By demonstrating the feasibility of this combined vaccine, our study contributes to the growing body of evidence supporting the use of multivalent, combined vaccines as a viable alternative to individual vaccines. The successful development of this formulation has the potential to simplify immunization schedules, reduce healthcare burdens, and improve global vaccination coverage, ultimately advancing public health outcomes. This work underscores the importance of continued innovation in vaccine design and highlights the critical role of preclinical studies in translating scientific advancements into practical solutions for disease prevention [23].

## 2. Materials and Methods

### 2.1. Materials

#### 2.1.1. Vaccine Preparation

The combined vaccine DTaP-Hib-MCV4 comprises three components: an adsorbed acellular pertussis, diphtheria, and tetanus toxoids vaccine (DTaP); a *Haemophilus influenzae* type b conjugate vaccine (Hib); and an ACYW135 meningococcal polysaccharide conjugate vaccine with CRM197 as the protein carrier (MCV4). Specifically, DTaP is adsorbed onto aluminum hydroxide, and aluminum phosphate adjuvants produced by CanSino Biologics Inc., with an aluminum (Al3+) content of 0.5 mg/mL. The Hib and ACYW135 meningococcal conjugated vaccines are formulated as a lyophilized preparation without an adjuvant. Each human dose (0.5 mL) contains 12.5 Lf diphtheria toxoid, 3.5 Lf tetanus toxoid, 25 μg per-tussis toxoid, 25 μg filamentous hemagglutinin (FHA), 8 μg pertactin (PRN), 10 μg *Haemophilus influenzae* type b capsular polysaccharide conjugated with TT, 10 μg meningococcal serogroup A capsular polysaccharide, 10 μg meningococcal serogroup C capsular polysaccharide, 5 μg meningococcal serogroup Y capsular polysaccharide, and 5 μg meningococcal serogroup W135 capsular polysaccharide individually conjugated with CRM197.

The DTaP vaccine is exactly the same as the combined vaccine’s DTaP component.

The Hib vaccine was prepared by covalently conjugating a purified capsular polysaccharide of *Haemophilus influenzae* type b (Hib) with tetanus toxoid. The active ingredient is 10 μg/dose of the Hib capsular polysaccharide without an adjuvant.

MCV4 vaccine is a commercial vaccine named Menhycia, produced by CanSino Biologics Inc., comprising two conjugate components: the group A and C meningococcal polysaccharide conjugate vaccine (CRM197 carrier) containing 10 μg of each polysaccharide (conjugated with CRM197) with sucrose and mannitol as excipients, and the group Y and W135 meningococcal polysaccharide conjugate vaccine (CRM197 carrier) containing 5 μg of each polysaccharide (conjugated with CRM197), formulated with sodium chloride, potassium hydrogen phosphate trihydrate, and potassium dihydrogen phosphate.

#### 2.1.2. Animal Models

Both male and female Sprague-Dawley (SD) rats aged 6–9 weeks were obtained from Zhejiang Vital River Laboratory Animal Technology Co., Ltd. for acute toxicity and repeated-dose toxicity studies, while female SD rats aged 4–6 weeks were sourced from Beijing Vital River Laboratory Animal Technology Co., Ltd. (Beijing, China) for an immunogenicity analysis. Female NIH mice weighing 12–14 g were obtained from Beijing Vital River Laboratory Animal Technology Co., Ltd. For an immunogenicity analysis. NIH mice, both male and female, weighing 10–12 g (M/F), 10–14 g (F), or 14–16 g (M/F), were procured from Beijing Vital River Laboratory Animal Technology Co., Ltd. For a potency test of DTaP. Pregnant SD rats at 18 days of gestation were also obtained from Beijing Vital River Laboratory Animal Technology Co., Ltd. for passive infant rat protection studies of Hib. All the animals were bred and maintained under specific pathogen-free (SPF) conditions. The animal study protocols were reviewed and approved by the institutional Animal Care and Uses Committee (IACUC) of Cansino Biologics.

#### 2.1.3. Bacteria Strains

The *Bordetella pertussis* bacterial strain, tetanus toxin reference materials, and diphtheria toxin reference materials were sourced from the National Institutes for Food and Drug Control (NIFDC); *Haemophilus influenzae* type b (Hib) strains were obtained from the National Center for Medical Culture Collections (NCMCC); meningococcal serogroup bacterial strains (A2909, C29026, Y29028, W29055, A2007056, CC1, YNM3131, W43744) were obtained from the Chinese Center for Disease Control and Prevention (China CDC, Beijing, China).

### 2.2. Methods

#### 2.2.1. Safety Analysis

##### Acute Toxicity Test

For the acute toxicity study completed by the JOINN (Suzhou) New Drug Research Center Co., Ltd. (Suzhou, China), 30 SD rats were randomly allocated into three groups (5 rats/sex/group), including a negative control group (saline) and two experimental groups receiving either 1 dose/rat or 3 doses/rat. Each rat received an intramuscular injection of either 0.5 mL (low-dose group) or 1.5 mL (high-dose group). Following administration, the rats were monitored for at least 4 h, followed by detailed weekly clinical observations and twice-daily general clinical observations for 14 consecutive days. Body weights were recorded upon arrival, before grouping, on the day prior to the first dose, and on days 3, 5, 7, and 14. Their food intake was assessed weekly. At the end of the observation period (day 15), all the animals were euthanized followed by systematic necropsy and gross observation. A histopathological examination was then performed on tissues/organs with gross anatomical abnormalities.

##### Repeated-Dose Toxicity Studies

In this experiment, also conducted by the JOINN (Suzhou) New Drug Research Center Co., Ltd., 150 SD rats (75 rats per sex) were randomly assigned to seven groups. groups 1–4 (15 rats per group per sex) served as the main test cohort, while groups 5–7 (5 rats per group per sex) were designated as the satellite group. Animals in groups 1 and 5 received 0.5 mL of physiological saline as the negative control. group 2 was administered with 0.5 mL of aluminum-containing adjuvants, while groups 3 and 6, and groups 4 and 7, were treated with DTaP-Hib-MCV4 at 1 dose per animal and 3 doses per animal, respectively, representing the low- and high-dose groups. Intramuscular injections were administered once every two weeks for eight consecutive weeks (five doses in total), followed by a four-week recovery period. The first 10 rats per sex per group in groups 1–4 were euthanized at the end of the eight-week treatment period (day 60), and the remaining animals were euthanized at the end of the recovery period (day 85).

A comprehensive set of evaluation parameters was employed, encompassing clinical observations, clinical pathology, immunology, and histopathology.

Clinical Assessments: Routine clinical observations were conducted, including regular monitoring of body weight, food intake, body temperature, and ophthalmic examinations.

Clinical Pathology: In the clinical pathology evaluations, blood cell count, coagulation function, blood biochemistry, and urine analysis were included.

Immunology: Immune Cell Phenotyping: Flow cytometry was used to quantify peripheral blood subsets of total T lymphocytes (CD3^+^), helper T cells (CD3^+^CD4^+^), and cytotoxic T cells (CD3^+^CD8^+^) using a BD FACSCanto™ II flow cytometer (Becton Dickinson^®^, Franklin Lakes, NJ, USA). The ratio of CD3^+^CD4^+^ to CD3^+^CD8^+^ was calculated. The fluorescently conjugated antibodies included FITC anti-rat CD3 antibody 1F4, APC anti-rat CD4 antibody W3/25, and PE anti-rat CD8a PE anti-rat antibody OX-8 (BioLegend^®^, San Diego, CA, USA).

Cytokine Analysis: Serum levels of proinflammatory cytokines, including tumor necrosis factor-alpha (TNF-α), interferon-gamma (IFN-γ), interleukin-2 (IL-2), and interleukin-6 (IL-6), were measured using a BD LSRFortessa™ flow cytometer (Becton Dickinson^®^) in conjunction with the LEGENDplex™ Rat Th Cytokine Panel (BioLegend^®^). The analysis utilized a set of commercial kits, including the LEGENDplex™ Rat IL-2/IL-6/IFN-γ/TN-α Capture Bead A7, 13X; LEGENDplex™ Rat Th Cytokine Panel Standard Set V02; LEGENDplex™ Rat Th Cytokine Panel Detection Abs V02; and LEGENDplex™ Buffer Set S.

Humoral Immunity: Serum-specific IgG antibodies were detected to evaluate the humoral immune response.

Histopathology: All the animals in groups 1–4 underwent necropsy. Organs were weighed, and a subsequent histopathological examination was performed.

#### 2.2.2. Immunogenicity Analysis

##### Immunization Procedure

A total of 40 National Institutes of Health (NIH) mice [18,19] or SD rats [17] were randomly assigned to five groups: the DTaP-Hib-MCV4 vaccine group, DTaP vaccine group, Hib vaccine group, MCV4 vaccine group, and saline control group. Each group received subcutaneous injections of 1/4 human doses of the respective vaccine formulation (based on an exploratory study of dose-dependent immunogenicity that identified 1/4HD as optimal, though this result was not included in the paper) on a 3-dose schedule at days 0, 14, and 28. Serum samples were collected 14 days after each administration, and antigen-specific antibody titers were quantified using an Enzyme-Linked Immunosorbent Assay (ELISA).

##### Detection of Serum-Specific IgG Antibodies by ELISA

Plastic microtiter plates (Nunc Maxisorp) were coated with specific antigens, including purified pertussis toxin (PT), filamentous hemagglutinin (FHA), pertactin (Prn), tetanus toxoid (TT), diphtheria toxoid (DT), Hib tyrosylated polysaccharides, and meningococcal group A, C, Y, and W135 polysaccharides, and incubated overnight at 4 °C. The following day, the plates were blocked with phosphate-buffered saline (PBS) containing 1% bovine serum albumin (BSA) at 37 °C for 1 h. Subsequently, 100 μL/well of 2-fold serially diluted serum samples were added to the plates and incubated at 37 °C for 1 h. After thorough washing, horseradish peroxidase (HRP)-conjugated goat anti-mouse (ZB-2305, Mfr. Beijing Zhong Shan-Golden Bridge Biological Technology Co., Ltd., Beijing, China) or anti-rat IgG antibodies (ZB-2307, Mfr. Beijing Zhong Shan-Golden Bridge Biological Technology Co., Ltd.) were added and incubated at 37 °C for 1 h. The colorimetric reaction was initiated by adding a 3,3′,5,5′-tetramethylbenzidine (TMB) substrate and stopped by adding 2 M of sulfuric acid (H_2_SO_4_). The optical density (OD) at 450 nm was measured using a microplate reader (BioTek, New York, NY, USA). Antibody titers were defined as the highest serum dilution ratio that yielded an OD_450nm_ value at least 2.1 times greater than the background value of the blank control.

#### 2.2.3. Protective Efficacy Analysis

##### Potency Test of DTaP

Potency testing serves as a cornerstone of vaccine quality control, providing rigorous evaluations of both immunological efficacy and batch-to-batch consistency. These evaluations may involve in vivo challenge models—such as exposing immunized mice to virulent *Bordetella pertussis* (*B. pertussis*) or lethal doses of tetanus toxin—to directly simulate pathogen interactions, with the protective efficacy quantified through survival rates. Alternatively, in vitro serological analyses, including diphtheria antitoxin neutralization assays using Vero cells, are employed to indirectly assess vaccine-induced humoral immunity by measuring toxin-neutralizing antibody titers in animal sera. Consequently, potency testing inherently functions as a comprehensive evaluation of protective efficacy, incorporating both direct pathogen challenge outcomes and indirect immunological correlates of protection [19].

##### Potency Test of Pertussis

Healthy NIH mice weighing 10–12 g, with an equal number of males and females, were randomly assigned to experimental groups. Three serial dilutions of the vaccine were prepared using a 5-fold dilution gradient in sterile saline, alongside a standard vaccine preparation. Each vaccine dilution was administered intraperitoneally to 20 mice at a volume of 0.5 mL. An unimmunized control group of 50 mice was divided into five subgroups of 10 mice each. After a 21-day observation period, all the immunized mice were challenged intracerebrally with a *Bordetella pertussis* bacterial suspension (80,000 bacteria/0.03 mL). The unimmunized control subgroups were challenged intracerebrally with 0.03 mL of bacterial suspensions containing 80,000, 8000, 800, 80, and 8 bacteria, respectively. The mice were monitored daily for 14 days. Deaths occurring before day 3 were excluded as non-significant (traumatic deaths). From day 4 to day 14, animals exhibiting symptoms such as paralysis, head swelling, arched back, or distinct hair erection were recorded as deaths. The results were statistically analyzed to determine the virulence (LD50) of the *B. pertussis* challenge suspension and to estimate potency of the tested vaccine. A statistical analysis of the results was performed using the Probit analysis method [19].

##### Potency Test of Tetanus Toxoid

NIH mice (14–16 g, with equal numbers of males and females) were randomly allocated into groups of 14 mice each, with an additional 10 mice serving as the control group. The reference standard vaccine was diluted in physiological saline at a ratio of 1:100. Both the reference standard vaccine and the test vaccine were subjected to four serial dilutions using a 2-fold dilution gradient. The mice were immunized subcutaneously with 0.5 mL of the respective vaccine dilutions. Twenty-eight days post-immunization, the mice were challenged subcutaneously with tetanus toxin (100 times the LD50 in a volume of 0.5 mL) and monitored for survival over the subsequent 5 days. The control group underwent an identical procedure but was challenged with twice the LD50 of tetanus toxin. A statistical analysis of the results was performed using the Probit analysis method [19].

##### Potency Test of Diphtheria Toxoid

Female NIH mice weighing 10–14 g were randomly assigned to experimental groups. The reference standard vaccine was diluted in physiological saline at a ratio of 1:24. Both the reference standard vaccine and the test vaccine were prepared in four serial dilutions using a 2-fold dilution gradient. Mice were immunized subcutaneously with 0.5 mL of the respective vaccine dilutions. Five weeks post-immunization, serum samples were collected for in vitro diphtheria antitoxin serum (DATS) neutralization potency tests using Vero cell assays. The diphtheria toxin concentration used in the assay was 1/10,000 Lcd. The results were assessed based on the color change of the cell culture medium: if DATS exhibited a low neutralization capacity, the cells would die, and the medium would remain red. Conversely, if the diphtheria toxin was neutralized, the Vero cells remained viable, and the medium turned yellow. The endpoint titer, expressed on a base-2 logarithmic scale, was defined as the reciprocal of the highest serum dilution that resulted in a yellow medium. A statistical analysis of the results was performed using the Parallel analysis method [19].

##### Passive Infant Rat Protection Studies of Hib

In this study, 5-day-old SD infant rats were used to evaluate the protective efficacy of DTaP-Hib-MCV4 and Hib vaccines against Hib strains. Thirty SD infant rats were randomly divided into three groups. Each group received a 0.1 mL subcutaneous injection of rat-derived serum from Hib- or DTaP-Hib-MCV4-immunized rats, administered near the dorsal neck region. In contrast, the control group was injected with negative rat serum. Four hours later, the animals were challenged intraperitoneally (i.p.) with 100 CFU of freshly cultured Hib (0.1 mL). Blood samples were collected 20 h post-challenge via cardiac puncture under Somnotol anesthesia and plated onto chocolate agar plates. Bacterial colonies were enumerated after 24 h by direct visual counting [20].

##### Serum Bactericidal Assay (SBA) of MCV4

Since the primary mechanism of *Neisseria meningitidis* clearance in humans involves specific antibody-dependent, complement-mediated bactericidal killing, functional antibody responses, as measured by the serum bactericidal assay (SBA), are currently employed to evaluate the efficacy of new meningococcal vaccines.

Meningococcal serogroup bacterial strains were streaked onto agar culture plates and incubated overnight at 37 °C in a 5% CO_2_ atmosphere to obtain isolated colonies. The strain was then subcultured by spreading it over the entire surface of another agar plate and incubating for 4 to 6 h at 37 °C with 5% CO_2_. Subsequently, the bacteria were suspended in D-PBS (Dulbecco’s PBS containing 0.5 mM MgCl_2_, 0.9 mM CaCl_2_, and 0.1% glucose; pH 7.4) to achieve an absorbance of 0.35 at 600 nm, using a cuvette with a 16 mm light path. Heat-inactivated (56 °C, 30 min) serum samples (50 μL) were serially diluted two-fold in buffer in 96-well, flat-bottom, tissue-culture-treated plates (Costar, Inc., Arlington, VA, USA). Equal volumes of bacterial suspension and pooled baby rabbit serum (BRS), serving as a complement source, were gently mixed, and 25 μL of the mixture was added to the serially diluted serum, followed by 5 min of shaking. The microtiter plate was then incubated at 37 °C without CO_2_ for 55 min. After incubation, 100 μL of a bacterial growth medium, tryptic soy broth (TSB), was added to each well, and the plate was incubated overnight at 37 °C with 5% CO_2_. The bactericidal titer for each serum sample was determined as the reciprocal of the serum dilution yielding ≥50% killing of the target bacteria compared to the pre-incubation bacterial count [21].

#### 2.2.4. Statistical Analysis

Antibody titers were expressed as geometric mean titers (GMT) with standard deviations (SDs). After logarithmic transformation using GraphPad software (version 8), statistical analyses and comparisons were conducted. For analyses involving two groups, t-tests (and nonparametric tests) were employed. When analyzing three or more groups, one-way ANOVAs (and nonparametric or mixed models) were used. Significant differences between groups were considered at *p*-values of 0.05, 0.01, and 0.001.

The data analysis for the long-term toxicity experiment was performed using SAS 9.4 statistical software, and the analysis methods are as follows: If the variance was homogeneous (*p* > 0.05), a one-way analysis of variance (ANOVA) was performed. If the ANOVA showed significant differences (*p* ≤ 0.05), Dunnett’s test was used for pairwise comparisons; if not, the data were logarithmically transformed (natural logarithm, Ln transformation) when there were no negative values within the groups, followed by reapplication of Levene’s test. If heterogeneity persisted after transformation or negative values were present (*p*≤ 0.05), the Kruskal–Wallis nonparametric test was applied to the original data. If the Kruskal–Wallis test showed significant differences (*p* ≤ 0.05), Mann–Whitney U (Wilcoxon) tests were used for pairwise comparisons.

## 3. Results

### 3.1. Safety Results

#### 3.1.1. Acute Toxicity Test

The acute toxicity test revealed no deaths, impending mortalities, or observable clinical signs in any group during the 14-day post-vaccination observation period.

A slight increase in body weight and a minor reduction in food intake were observed in the one-dose and the three-dose groups starting on day 3 post administration compared to the sex-matched negative control group. The animals in the one-dose and the three-dose groups showed slower weight gain, starting from D3, compared to the same-sex negative control group of the same period (with the maximum reduction of 6.1%), indicating a time- and dose-dependent response relationship, and the changes were consistent in both male and female animals. Meanwhile, the food intake slightly decreased within one week after administration (with the maximum reduction of 9.7%) and recovered by two weeks after administration, suggesting a correlation with the administration time. These two phenomena are considered to be possibly related to local irritation caused by the administration.

At necropsy on day 15, nodules at the local injection site were observed in 4 out of 10 animals (two males and two females in the one-dose group, and one male and three females in the three-dose group). The gross observation showed nodules, and the microscopic examination revealed granulomatous inflammation, likely associated with the aluminum adjuvant in the vaccine formulation. In conclusion, under the conditions of this study, the combined vaccine resulted in slow weight gain, a slight decrease in food intake, and as well as localized pathological changes attributable adjuvant-related limitations. The maximum tolerated dose (MTD) was determined to be at least three doses per rat.

#### 3.1.2. Repeated-Dose Toxicity Studies

Throughout the repeated-dose toxicity studies, no fatalities, impending mortalities, or abnormalities in clinical physiology were observed. Additionally, no abnormalities were detected in body temperature, ophthalmic examination, urinalysis, immune cell phenotypic analysis (Supplementary Information Table S1), cytokine levels (Supplementary Information Table S2), or organ weights (Supplementary Information Table S3).

Significant changes were observed in the following indicators during the trial:

Body weight and food intake in the high-dose group decreased 1 week after each administration, with the reductions ranging from 15.4% to 44.6% and 1.2% to 10.3%, respectively. Recovery was observed after the discontinuation of the vaccine (weeks 2, 4, 6, and 8), which was attributed to local irritation following high-dose administration.

Blood Cell Count: At day 60 (3 days after the last dosing), increases in neutrophils (Neut; excluding females in the adjuvant control group), monocytes (Mono), and eosinophils (Eos) were observed in the adjuvant control group, low-dose group, and high-dose group. White blood cell (WBC) counts were elevated in the low- and high-dose vaccine groups at day 60, with a greater magnitude of changes compared to the adjuvant control group at the same adjuvant dose level, likely related to local inflammatory responses at the injection site and/or immune responses induced by the vaccine. All the groups showed slight decreases in hemoglobin (Hb), hematocrit (Hct), and mean corpuscular hemoglobin (MCH) at day 60. Reticulocyte counts decreased (except male rats in the adjuvant control group) at day 4 (3 days after the first dosing), with more pronounced changes in vaccine groups than the adjuvant control group, attributed to acute-phase responses and/or immune responses from the vaccine/adjuvant (Supplementary Information Table S4). All the indices recovered by day 85 (4 weeks post-withdrawal).

Coagulation Function: Fibrinogen (FIB) elevation occurred in the adjuvant control group (excluding males at day 4 and females at day 60), low-dose group, and high-dose groups within 1–3 days after the first and last dosing, associated with acute-phase responses. The high-dose group showed more significant changes than the adjuvant control group at the same dose, suggesting the involvement of vaccine-induced immune responses (Supplementary Information Table S5). All the alterations resolved by day 85.

Blood Biochemistry: At day 60, decreases in albumin (Alb) and albumin/globulin ratio (A/G) were observed in the adjuvant control, low-dose group, and high-dose group. The high-dose vaccine group exhibited greater changes than the adjuvant control group at the same dose, attributed to acute-phase responses and potential vaccine-induced immune effects (Supplementary Information Table S6). These parameters normalized by day 85.

Histopathology: At day 60, histopathological examination revealed mild-to-marked granulomatous inflammation at the intramuscular injection site in the adjuvant control group, low-dose group, and high-dose groups (Supplementary Information Table S7). These observed lesions were directly attributed to the vaccines or the adjuvant control. Following a four-week recovery period (day 85), granulomatous inflammation persisted but exhibited reduced severity: inflammation at the injection site decreased from mild to moderate, while in the biceps femoris muscle, it subsided from minimal to mild, indicating partial lesion resolution. Notably, all the epimysial lesions in the biceps femoris had completely resolved, suggesting a significant recovery response over the four-week period.

Serum-specific IgG binding antibodies: In both the low-dose and high-dose groups, antibodies against PT, FHA, PRN, DT, TT, Hib, and A, C, Y, and W135 polysaccharides were detectable 2 weeks after the first dose (prior to the second dose). The highest antibody titers ranged from 1:200 to >1:437,400. Furthermore, antigen-specific antibody titers increased with the number of immunizations, as indicated by ELISA, demonstrating a significant enhancement effect. The peak titers remained stable until 4 weeks after the final vaccination (day 85), with only minor reductions observed in anti-Hib and anti-C IgG antibody levels.

### 3.2. Immunogenicity Results

#### 3.2.1. Immunogenicity of DTaP-Hib-MCV4 Vaccine in a Mouse Model

**Figure 1:**
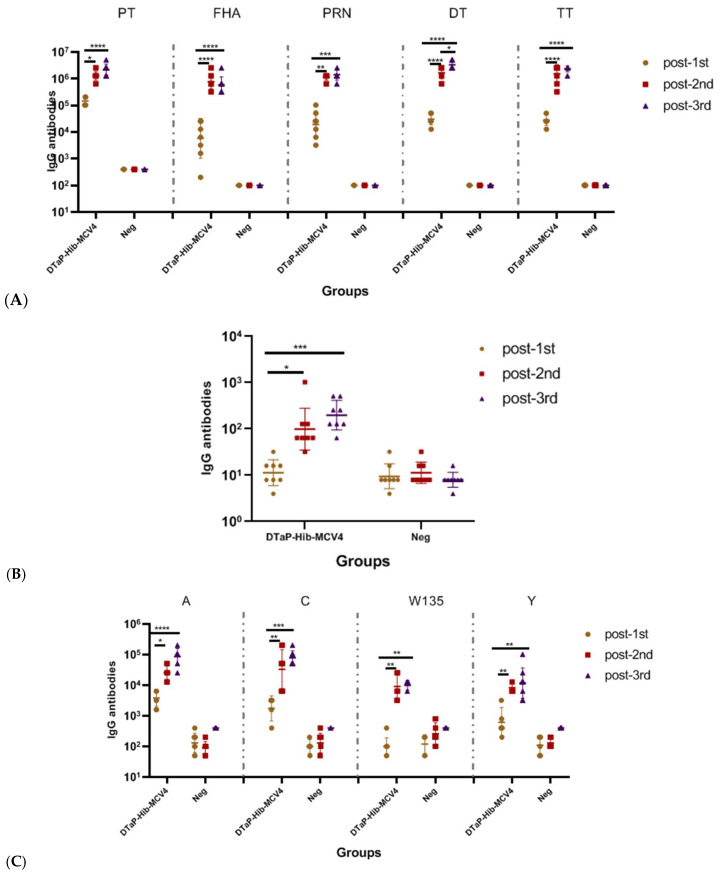
Humoral immune responses of the DTaP-Hib-MCV4 vaccine administered with different doses to NIH mice via the subcutaneous (s.c.) immunization route. The NIH mice were immunized with DTaP-Hib-MCV4 on days 0, 14, and 28, and blood samples were collected on days 14, 28, and 42 before the next vaccine. Serum IgG binding antibodies against DTaP (**A**), Hib (**B**), and MCV4 (**C**) were measured by ELISA. The data represents the geometric mean titer (GMT) ± standard deviation (SD), *n* = 8 in each group. *p* values of ≥0.05 indicate no significant difference. When there was no significant difference between the two groups, it was not marked in the picture. * *p* < 0.05, ** *p* < 0.01, *** *p* < 0.001, **** *p* < 0.0001.

The humoral immunogenicity of the DTaP-Hib-MCV4 vaccine administered at different time points was first assessed in an NIH mouse model, using 1/4 of the human dose (HD) per inoculation (Figure 1). Following the second booster vaccination, robust antibody responses were observed against pertussis (anti-PT IgG GMT = 1,280,000; anti-FHA IgG GMT = 761,093; anti-PRN IgG GMT = 1,159,326), diphtheria toxoid (anti-DT IgG GMT = 1,659,955), tetanus toxoid (anti-TT IgG GMT = 1,522,185), Hib (anti-Hib IgG GMT = 99), and ACYW135 group meningococcus (anti-A IgG GMT = 25,600; anti-C IgG GMT = 33,199; anti-Y IgG GMT = 8300; anti-W135 IgG GMT = 9051). Additionally, IgG titers against DT were further upregulated after the third vaccination. Overall, two doses of the DTaP-Hib-MCV4 vaccine were sufficient to induce strong humoral immune responses.

#### 3.2.2. The Immunogenicity of the DTaP-Hib-MCV4 Vaccine in a Rat Model

**Figure 2:**
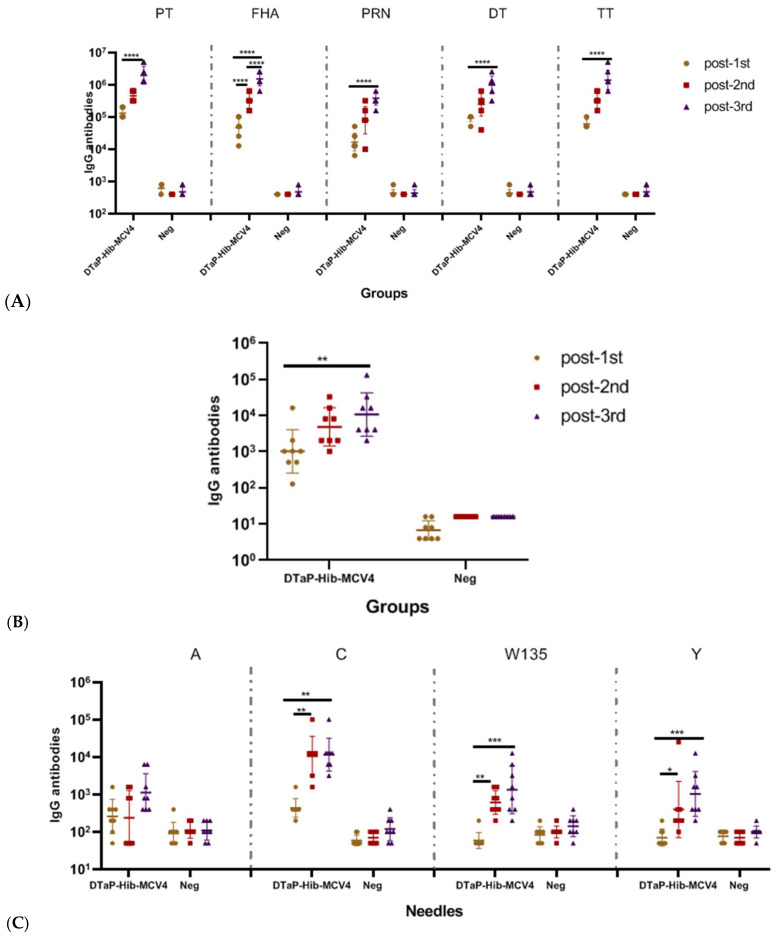
Humoral immune responses to the DTaP-Hib-MCV4 vaccine at different doses in SD rats following s.c. immunization. The SD rats were immunized with DTaP-Hib-MCV4 on days 0, 14, and 28, and blood samples were collected on day 14, 28, and 42 (prior to the next vaccination). Serum IgG binding antibodies against the DTaP group (**A**), Hib (**B**), and MCV4 (**C**) were measured by ELISA. The data presents as the geometric mean titer (GMT) ± standard deviation (SD), *n* = 8 in each group. *p* values of ≥0.05 indicate no significant difference. When there was no significant difference between the two groups, it was not marked in the picture. * *p* < 0.05, ** *p* < 0.01, *** *p* < 0.001, **** *p* < 0.0001.

The immunogenicity of the DTaP-Hib-MCV4 vaccine was further evaluated in an SD rat model. Unlike the IgG titer plateau observed after the second booster in NIH mice, the third vaccination in SD rats generally resulted in a continued increase in antibody responses, particularly for DTaP-associated antigens. Specific IgG titers against pertussis (anti-PT IgG GMT = 1,974,030; anti-FHA IgG GMT = 1,522,185; anti-PRN IgG GMT = 380,546), diphtheria toxoid (anti-DT IgG GMT = 987,015), tetanus toxoid (anti-TT IgG GMT = 1,395,850), Hib (anti-Hib IgG GMT = 10,624), and ACYW135 group meningococcus (anti-A IgG GMT = 1131; anti-C IgG GMT = 11,738; anti-Y IgG GMT = 1037; anti-W135 IgG GMT = 1345) are presented in Figure 2.

#### 3.2.3. Immunogenicity of DTaP-Hib-MCV4 Combination Vaccine vs. Each Individual Vaccine in Murine Models

To address whether combination vaccines provide enhanced immunogenicity compared to individual vaccines, we conducted a comparative evaluation of the immunogenicity of the DTaP-Hib-MCV4 combination vaccine against DTaP, Hib, and MCV4 individual vaccines in both NIH mice and SD rat models.

**Figure 3:**
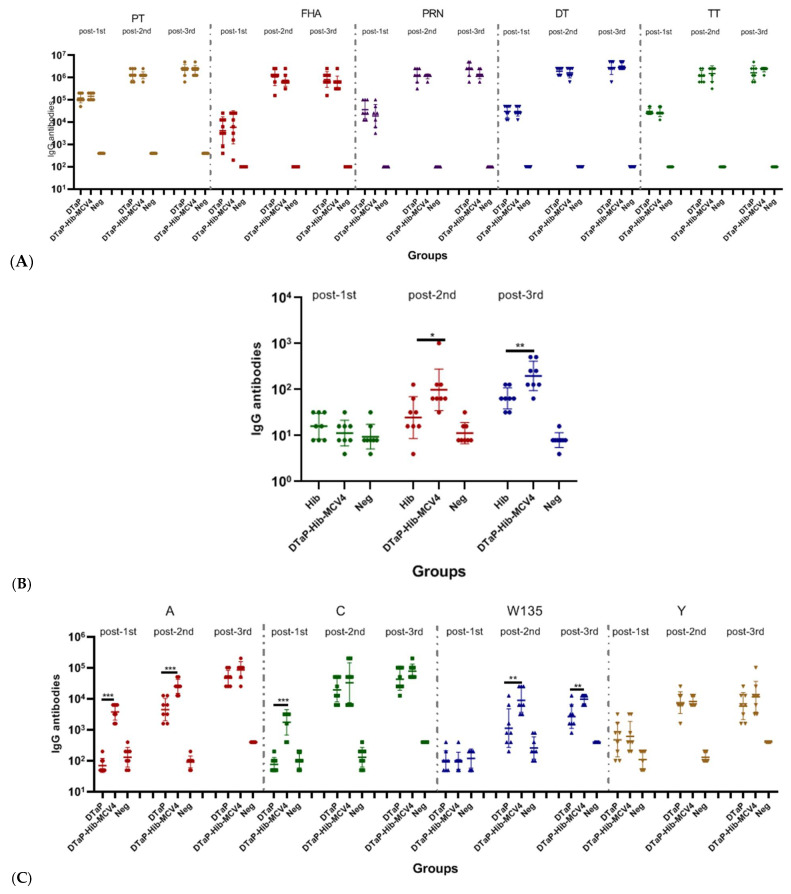
Humoral immune responses to the DTaP-Hib-MCV4 vaccine compared with those induced by DTaP, Hib, and MCV4 individual vaccines in an NIH mice model via s.c. immunization. The NIH mice were immunized with DTaP-Hib-MCV4, DTaP, Hib, or MCV4 on days 0, 14, and 28, and blood samples were collected on days 14, 28, and 42 (prior to the next vaccination). Serum IgG binding antibodies against DTaP group (**A**), Hib (**B**), and MCV4 (**C**) were measured by ELISA. The data are presented as the geometric mean titer (GMT) ± standard deviation (SD), *n* = 8 in each group. *p* values of ≥ 0.05 indicate no significant difference. When there was no significant difference between the two groups, it was not marked in the picture. * *p* < 0.05, ** *p* < 0.01, *** *p* < 0.001.

NIH mice were administered with three doses of either the DTaP-Hib-MCV4 vaccine or the DTaP, Hib, and MCV4 individual vaccines. Blood samples were collected 14 days after each immunization to measure serum IgG titers. As shown in Figure 3, the immunogenicity of the DTaP-Hib-MCV4 vaccine was not inferior and, in some cases, it was superior to that of the individual vaccines. For example, it demonstrated stronger immune responses for Hib-specific antibodies (IgG GMT = 99 vs. 25 after two doses and 197 vs. 64 after three doses). Additionally, the DTaP-Hib-MCV4 vaccine induced significantly higher IgG responses against group A and W135 meningococcus (anti-A IgG GMT = 25,600; anti-W135 IgG GMT = 9051) compared to the MCV4 vaccine (anti-A IgG GMT = 4525; anti-W135 IgG GMT = 1131) after two doses. Furthermore, W135-specific IgG levels were further enhanced after three doses, with IgG GMT values of 9870 and 2691 for the DTaP-Hib-MCV4 and MCV4 vaccines, respectively.

**Figure 4:**
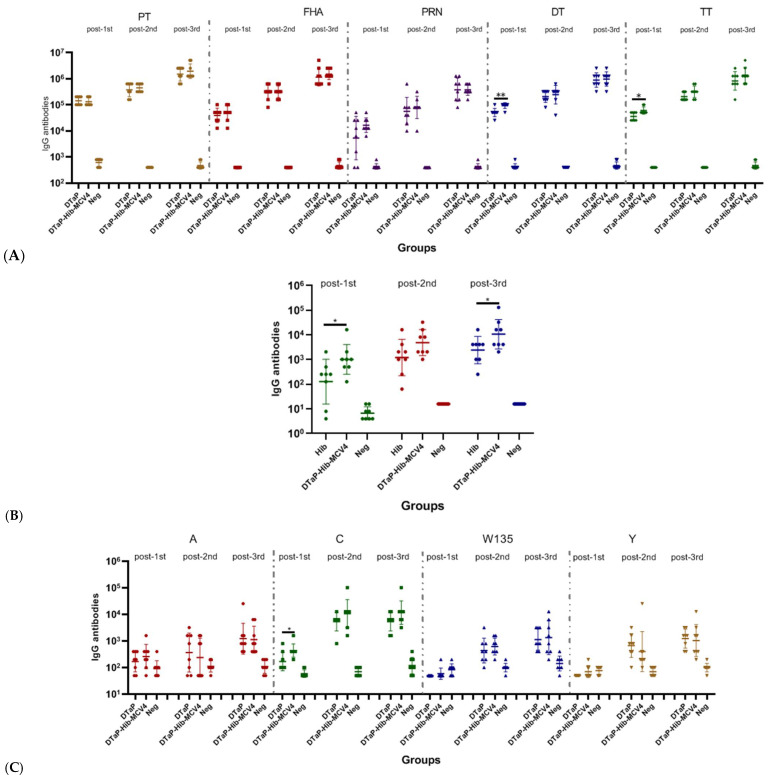
The humoral immune response to the DTaP-Hib-MCV4 vaccine compared with DTaP, Hib, and MCV4 individual vaccines in an SD rat model via s.c. immunization. The SD rats were immunized with DTaP-Hib-MCV4, DTaP, Hib, or MCV4 on days 0, 14, and 28, and blood samples were collected on days 14, 28, and 42 (prior to the next vaccination). Serum IgG binding antibodies against the DTaP group (**A**), Hib (**B**), and MCV4 (**C**) were measured by ELISA. The data are represented as the geometric mean titer (GMT) ± standard deviation (SD), *n* = 8 in each group. *p* values of ≥0.05 indicate no significant difference. When there was no significant difference between the two groups, it was not marked in the picture. * *p* < 0.05, ** *p* < 0.01.

A similar procedure was followed to assess immunogenicity in SD rats. The IgG responses induced by the DTaP-Hib-MCV4 vaccine were not inferior to those elicited by the individual vaccines (Figure 4). Notably, a few significant increases in antibody levels were observed after a single dose of the DTaP-Hib-MCV4 vaccine compared to the individual vaccines. For instance, the DTaP-Hib-MCV4 vaccine elicited stronger DT- and TT-specific antibody responses (anti-DT IgG GMT = 93,901; anti-TT IgG GMT = 60,887) than the DTaP vaccine (anti-DT IgG GMT = 51,200; anti-TT IgG GMT = 36,204).

### 3.3. Protective Efficacy Results

#### 3.3.1. Potency Test of DTaP

**Figure 5:**
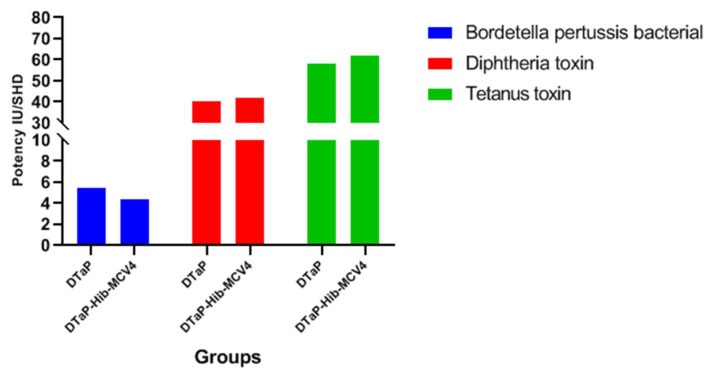
Results of potency test of DTaP vs. DTaP-Hib-MCV4. (IU/SHD = International Unit/Standard Human Dose).

The potency of the diphtheria–tetanus–acellular pertussis (DTaP) component within the DTaP-Hib-MCV4 combination vaccine was quantitatively assessed and tabulated (Figure 5). The results confirmed full compliance with the potency specifications outlined in the Chinese Pharmacopoeia (ChP), demonstrating a robust protective efficacy against *Bordetella pertussis*, tetani toxin, and diphtheriae toxoid. Critically, these data validate adherence to the regulatory principle for combination vaccines: “the potency of each antigenic constituent must meet or exceed the established thresholds defined for its corresponding individual formulation”. This ensures immunological compatibility and the absence of antigenic interference within the multicomponent vaccine system.

#### 3.3.2. Passive Infant Rat Protection Studies of Hib

The immunoprotective efficacy of the rat anti-Hib antiserum was evaluated through the utilization of an infant rat bacteremia model. Specifically, infant rats that were 5 days old were subcutaneously injected with anti-Hib rat antisera, which were obtained from rats that had been immunized with the DTaP-Hib-MCV4 vaccine. Meanwhile, the control groups were injected with a rat negative serum. After a period of 24 h, these animals were then challenged with Hib CMCC (at a dosage of 100 CFU). Subsequently, blood samples were collected 20 h after the challenge and were inoculated onto chocolate agar. Twenty-four hours later, the bacterial colonies were counted to evaluate the occurrence of bacteremia. Remarkably, none of the 10 animals that received the rat anti-Hib antiserum sourced from the DTaP-Hib-MCV4 vaccine demonstrated any signs of bacteremia following the challenge. In stark contrast, all 10 infant rats in the negative antiserum group developed bacteremia, with an average bacterial count of 5814 CFU per 0.1 mL of blood. These results incontrovertibly demonstrate that the passive transfer of anti-Hib antibodies was effective in safeguarding the infant rats against the Hib challenge, thereby indicating that the DTaP-Hib-MCV4 vaccine is capable of eliciting functional anti-Hib antibodies. Furthermore, among the 10 animals that were administered rat anti-Hib antiserum from the Hib vaccination group, only 1 animal exhibited bacteremia after being challenged with live Hib CMCC (with a bacterial count of 0.7 CFU per 0.1 mL). When combined with the results obtained from the DTaP-Hib-MCV4 vaccine group, these findings suggest that the functional anti-Hib antibodies induced by the DTaP-Hib-MCV4 vaccine did not experience any impairment as a result of being a part of the combined vaccine formulation.

#### 3.3.3. Serum Bactericidal Assay (SBA) of MCV4

**Figure 6:**
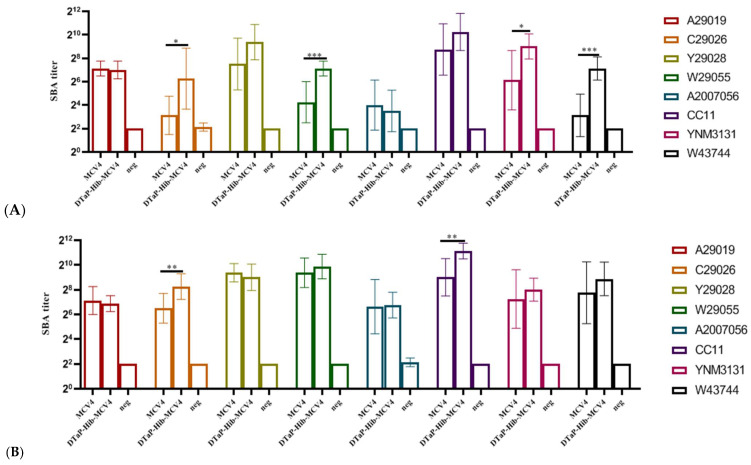
The SBA response to the DTaP-Hib-MCV4 vaccine and the MCV4 vaccine after three doses of immunization. NIH mice (**A**) and SD rats (**B**) were immunized with DTaP-Hib-MCV4 or MCV4 on days 0, 14, and 28, and blood samples were collected on day 42. The data are represented as the geometric mean titer (GMT) ± standard deviation (SD), *n* = 8 in each group. *p* values of ≥0.05 indicate no significant difference. When there was no significant difference between the two groups, it was not marked in the picture. * *p* < 0.05, ** *p* < 0.01, *** *p* < 0.001.

The immunoprotective efficacy of the anti-MCV4 antiserum was evaluated using serum bactericidal assays (SBA) in mouse and rat models (Figure 6). The results demonstrated that the DTaP-Hib-MCV4 vaccine elicited functional bactericidal antibodies against serogroups A, C, Y, and W135 in both the murine models (anti-A29019 GMT = 128; anti-C29026 GMT = 76; anti-Y29028 GMT = 664; anti-W29055 GMT = 140; anti-A2007056 GMT = 11; anti-CC1 GMT = 1218; anti-YNM3131 GMT = 512; anti-W43744 GMT = 140) and the rat models (anti-A29019 GMT = 117; anti-C29026 GMT = 304; anti-Y29028 GMT = 512; anti-W29055 GMT = 939; anti-A2007056 GMT = 108; anti-CC1 GMT = 2233; anti-YNM3131 GMT = 256; anti-W43744 GMT = 470). These results conclusively demonstrate that the DTaP-Hib-MCV4 vaccine induces the production of functional anti-MCV4 antibodies.

Moreover, the functional bactericidal antibodies produced by the DTaP-Hib-MCV4 vaccine were not diminished compared to those induced by the MCV4 vaccine alone; in fact, some bactericidal antibody titers were even higher. These findings indicate that the functional anti-MCV4 antibodies induced by the DTaP-Hib-MCV4 vaccine were not adversely affected by the vaccine combination.

## 4. Discussion

The development of combined vaccines represents a pivotal strategy to address the increasing complexity of immunization schedules and enhance global vaccination coverage. This study provides comprehensive preclinical data on the safety, immunogenicity, and protective efficacy of a novel combined vaccine, DTaP-Hib-MCV4, which integrates diphtheria, tetanus, acellular pertussis, *Haemophilus influenzae* type b (Hib), and ACYW135 meningococcal conjugate components. Our findings demonstrate that the combined vaccine is well-tolerated, immunogenic, and capable of eliciting robust protective immune responses, supporting its potential as a viable alternative to individual vaccines.

The acute and repeated-dose toxicity studies in Sprague-Dawley (SD) rats showed that the DTaP-Hib-MCV4 vaccine was well-tolerated, with no significant systemic toxicity observed. In the toxicological experimental design, a 1.5 mL high-dose group was included. This design follows toxicology test principles aiming to induce obvious toxic reactions in animals for safety margin evaluation [24,25]. The acute and repeated-dose toxicity studies in Sprague-Dawley (SD) rats further demonstrated that the DTaP-Hib-MCV4 vaccine was well-tolerated at the MTD of 1.5 mL, with no significant systemic toxicity detected. Local reactions, such as granulomatous inflammation at the injection site, were consistent with the expected effects of the aluminum adjuvant and aligned with previous reports for other aluminum-adjuvanted vaccines [26,27]. These findings are consistent with the safety profiles of established combined vaccines, such as DTaP-Hib and DTaP-IPV-Hib, which have been widely implemented in pediatric immunization programs [6,28,29]. Meanwhile, in the statistical data, the thymus weight of the male control group was significantly smaller than that of the vaccine group, while the female control group showed a significantly higher thymus weight than the vaccine group. However, when comparing the thymus weights among all the experimental groups, the differences were not statistically significant. Additionally, there were no pathological abnormalities, and no dose-response relationship was observed. We speculate that this might be due to the small size of the thymus in rats and the individual variations among them.

The combined vaccine elicited robust humoral immune responses in both SD rats and NIH mice. Antibody titers against all the vaccine components reached levels comparable to or exceeding those induced by each individual vaccine. Notably, the third dose of the vaccine further enhanced antibody responses, particularly for the DTaP components, suggesting that the combined formulation does not compromise immunogenicity. These results are consistent with prior studies demonstrating that multivalent vaccines can induce synergistic immune responses without significant antigenic interference [30,31,32].

In the DTaP efficacy test, the vaccine was diluted with normal saline, which is a clear stipulation in the Chinese Pharmacopoeia [19]. Theoretically, normal saline might affect the particle size and adsorption of the antigen. However, our research data indicate that normal saline did not affect the vaccine’s efficacy, and the results were reproducible. Potency tests for the DTaP components confirmed that the combined vaccine met pharmacopoeia standards, providing effective protection against pertussis, tetanus, and diphtheria. Passive infant rat protection studies demonstrated that the vaccine-induced anti-Hib antibodies were functional, effectively preventing bacteremia in challenged animals. Similarly, serum bactericidal assays (SBA) for the MCV4 components confirmed the induction of functional antibodies against meningococcal serogroups A, C, Y, and W135. These findings align with the protective efficacy observed in other combined vaccines, such as DTaP-IPV-Hib and MenACWY conjugate vaccines [33,34,35].

The primary advantage of the DTaP-Hib-MCV4 vaccine lies in its ability to reduce the number of injections required for immunization, thereby simplifying vaccination schedules and improving adherence. This is particularly significant in low- and middle-income countries [36], where logistical challenges and limited healthcare resources often hinder vaccination coverage. Additionally, the combined vaccine has the potential to reduce healthcare costs and minimize the risk of the adverse events associated with multiple injections [37].

While the preclinical data are promising, several limitations must be acknowledged. First, the study was conducted in animal models, and the results may not fully translate to human populations. Future clinical trials are necessary to confirm the safety, immunogenicity, and protective efficacy of the DTaP-Hib-MCV4 vaccine in humans [38]. Second, the study did not evaluate the long-term durability of immune responses or the potential for immune interference between vaccine components over time. Further studies are warranted to assess the persistence of immunity and the potential need for booster doses [39]. Finally, the study focused primarily on humoral immunity, and the role of cellular immune responses in protection against these pathogens remains to be explored [40], representing a critical gap to be addressed in future mechanistic investigations.

To address these gaps, future research must follow a systematic framework. While the preclinical data provide a strong foundation for further development, human clinical trials are indispensable to confirm safety, immunogenicity, and efficacy in the human population. If successfully translated, the DTaP-Hib-MCV4 vaccine could emerge as a significant milestone in pediatric immunization, addressing the public health burdens posed by vaccine-preventable diseases and contributing to global initiatives aimed at achieving universal health coverage [41]. Future research should prioritize characterizing long-term immune memory and mechanistic pathways of action to facilitate the vaccine’s eventual regulatory approval and widespread implementation.

## 5. Conclusions

In conclusion, the DTaP-Hib-MCV4 combined vaccine demonstrated an acceptable safety profile, robust immunogenicity, and definitive protective efficacy in preclinical investigations. By integrating multiple antigens into a single formulation, it presents a pragmatic solution to streamline immunization protocols, particularly in regions facing challenges in vaccine delivery, and holds substantial promise for improving global vaccination equity and coverage.

## Data Availability

The datasets of this study are available from the corresponding author upon reasonable request.

## Supplementary Materials

The following supporting information can be downloaded at https://www.mdpi.com/article/10.3390/vaccines13070724/s1, Table S1: Immune cell phenotypic analysis; Table S2: Cytokine analysis (pg/mL); Table S3: Statistical data of organ weights (D60); Table S4: Statistical data of blood cell count; Table S5: Statistical data of coagulation function; Table S6: Statistical data of blood biochemistry; Table S7: The tissues that were examined by light microscopy.
