# Supplementary material for: An Evaluation of the Safety, Immunogenicity, and Protective Efficacy of a Combined Diphtheria–Tetanus–Acellular Pertussis, Haemophilus influenzae Type b, and ACYW135 Meningococcal Conjugate Vaccine in Murine and Rat Models
Source: Vaccines (Basel). 2025 Jul 3;13(7):724. doi: 10.3390/vaccines13070724 (PMC12299065; doi:10.3390/vaccines13070724)
Supplement: Supplementary file 1 [file vaccines-13-00724-s001.zip › vaccines-3692406-supplementary.pdf]

Supplementary Information

Table S1. Immune cell phenotypic analysis.

Sex: M		Immune Cell Phenotypes					
		CD3+ (%) [g]	CD3+ (%) [g]	CD3+CD4+ (%) [g]	CD3+CD4+ (%) [g]	CD3+CD8+ (%) [g]	CD3+CD8+ (%) [g]
		D60	D85	D60	D85	D60	D85
Normal saline control group	Mean	40.22	27.46	54.74	49.94	39.32	42.08
	SD	4.17	3.67	12.87	11.22	11.25	9.73
	N	5	5	5	5	5	5
Adjuvant control group	Mean	39.40	24.14	61.58	51.88	32.74	37.26
	SD	7.17	3.52	6.55	10.16	7.39	7.30
	N	5	5	5	5	5	5
	%Diff	-2.0	-12.1	12.5	3.9	-16.7	-11.5
0.5mL-dose group	Mean	38.94	29.80	55.46	58.42	38.50	36.12
	SD	8.73	7.98	6.48	5.86	5.36	4.47
	N	5	5	5	5	5	5
	%Diff	-3.2	8.5	1.3	17.0	-2.1	-14.2
1.5mL-dose group	Mean	36.82	29.78	62.88	50.52	31.68	43.44
	SD	8.12	5.04	5.89	5.98	6.26	4.55
	N	5	5	5	5	5	5
	%Diff	-8.5	8.4	14.9	1.2	-19.4	3.2

[g] - ANOVA & Dunnett

Sex: M		Immune Cell Phenotypes	
		CD3+CD4+/ CD3+CD8+ [g]	CD3+CD4+/ CD3+CD8+ [g]
		D60	D85
Normal saline control group	Mean	1.656	1.285
	SD	1.168	0.542
	N	5	5
Adjuvant control group	Mean	2.032	1.503
	SD	0.840	0.713
	N	5	5
	%Diff	22.7	17.0
0.5mL-dose group	Mean	1.481	1.654
	SD	0.380	0.368
	N	5	5
	%Diff	-10.5	28.7
1.5mL-dose group	Mean	2.067	1.183
	SD	0.536	0.252
	N	5	5
	%Diff	24.8	-7.9

[g] - ANOVA & Dunnett

Sex: F		Immune Cell Phenotypes					
		CD3+ (%) [g]	CD3+ (%) [g1]	CD3+CD4+ (%) [g2]	CD3+CD4+ (%) [g2]	CD3+CD8+ (%) [g2]	CD3+CD8+ (%) [g2]
		D60	D85	D60	D85	D60	D85
Normal saline control group	Mean	52.22	38.74	57.38	52.00	36.08	40.18
	SD	5.89	5.73	3.90	7.34	2.64	6.57
	N	5	5	5	5	5	5
Adjuvant control group	Mean	49.12	34.80	61.46	49.54	29.60	39.84
	SD	6.81	7.02	4.00	1.93	3.07	3.46
	N	5	5	5	5	5	5
	%Diff	-5.9	-10.2	7.1	-4.7	-18.0	-0.8
0.5mL-dose group	Mean	48.48	41.58	56.50	53.56	36.98	37.98
	SD	5.70	13.94	6.58	10.36	5.98	5.23
	N	5	5	5	5	5	5
	%Diff	-7.2	7.3	-1.5	3.0	2.5	-5.5
1.5mL-dose group	Mean	42.92	39.50	62.82	58.12	31.42	33.74
	SD	6.54	7.02	8.12	10.36	4.40	8.63
	N	5	5	5	5	5	5
	%Diff	-17.8	2.0	9.5	11.8	-12.9	-16.0

[g2] - ANOVA & Dunnett; [g] - ANOVA & Dunnett; [g1] - ANOVA & Dunnett (Log)

Sex: F		Immune Cell Phenotypes	
		CD3+CD4+/ CD3+CD8+ [g]	CD3+CD4+/ CD3+CD8+ [g]
		D60	D85
Normal saline control group	Mean<br>SD<br>N	1.602<br>0.206<br>5	1.341<br>0.367<br>5
Adjuvant control group	Mean<br>SD<br>N<br>%Diff	2.098<br>0.285<br>5<br>31.0	1.250<br>0.107<br>5<br>-6.8
0.5mL-dose group	Mean<br>SD<br>N<br>%Diff	1.587<br>0.463<br>5<br>-0.9	1.468<br>0.524<br>5<br>9.4
1.5mL-dose group	Mean<br>SD<br>N<br>%Diff	2.054<br>0.498<br>5<br>28.2	1.895<br>0.882<br>5<br>41.3

[g] - ANOVA & Dunnett

Note: All percentages (%) denoted the proportion of CD3+, CD3+CD4+ and CD3+CD8+cells relative to total lymphocytes.

Table S2. Cytokine analysis (pg/mL).

Sex: M		Cytokines						
		IL-2 [I]	IL-2 [I]	IL-2 [I]	IL-6 [g]	IL-6 [I]	IL-6 [I]	TNF-α [I]
		D4	D60	D85	D4	D60	D85	D4
Normal saline control group	Mean<br>SD<br>N	146.915 n<br>52.715<br>2	212.280 n<br>.<br>1	. n<br>.<br>0	431.176<br>98.052<br>5	410.400 n<br>.<br>1	380.455 n<br>42.349<br>2	. n<br>.<br>0
Adjuvant control group	Mean<br>SD<br>N<br>%Diff	257.885 n<br>84.209<br>2<br>75.5	. n<br>.<br>0<br>.	134.490 n<br>43.041<br>3<br>.	677.295<br>318.350<br>4<br>57.1	400.365 n<br>14.192<br>2<br>-2.4	400.665 n<br>60.230<br>4<br>5.3	153.440 n<br>17.508<br>2<br>.
0.5mL-dose group	Mean<br>SD<br>N<br>%Diff	146.060 n<br>73.935<br>2<br>-0.6	. n<br>.<br>0<br>.	139.427 n<br>45.211<br>3<br>.	481.390 n<br>71.884<br>2<br>11.6	370.487 n<br>34.370<br>3<br>-9.7	569.050 n<br>95.194<br>5<br>49.6	. n<br>.<br>0<br>.
1.5mL-dose group	Mean<br>SD<br>N<br>%Diff	109.640 n<br>.<br>1<br>-25.4	. n<br>.<br>0<br>.	183.828 n<br>84.952<br>5<br>.	532.220 n<br>.<br>1<br>23.4	. n<br>.<br>0<br>.	634.412 n<br>80.399<br>5<br>66.8	. n<br>.<br>0<br>.

[I] - n - Inappropriate for statistics; [g] - ANOVA & Dunnett: n - Inappropriate for statistics

Sex: M		Cytokines				
		TNF-α [I]	TNF-α [I]	IFN-γ [g]	IFN-γ [I]	IFN-γ [I]
		D60	D85	D4	D60	D85
Normal saline control group	Mean<br>SD<br>N	. n<br>.<br>0	. n<br>.<br>0	165.950<br>36.411<br>5	241.800n<br>.<br>1	. n<br>.<br>0
Adjuvant control group	Mean<br>SD<br>N<br>%Diff	. n<br>.<br>0<br>.	. n<br>.<br>0<br>.	293.643<br>147.200<br>4<br>76.9	7509.230n<br>.<br>1<br>3005.6	2042.115 n<br>3734.084<br>4<br>.

0.5mL-dose group	Mean	. n	. n	159.180	. n	183.715 n
	SD	.	.	43.311	.	68.495
	N	0	0	4	0	4
	%Diff	.	.	-4.1	.	.
1.5mL-dose group	Mean	. n	128.770 n	159.490 n	. n	301.628 n
	SD	.	.	.	.	58.473
	N	0	1	1	0	5
	%Diff	.	.	-3.9	.	.

[I] - n - Inappropriate for statistics; [g] - ANOVA & Dunnett(Log): n - Inappropriate for statistics

Sex: F		Cytokines						
		IL-2 [I]	IL-2 [I]	IL-2 [I]	IL-6 [I]	IL-6 [I]	IL-6 [g]	TNF-α [I]
		D4	D60	D85	D4	D60	D85	D4
Normal saline control group	Mean	77.510 n	212.280 n	109.640 n	410.795 n	481.390 n	431.330	. n
	SD	.	.	.	85.256	71.884	123.186	.
	N	1	1	1	2	2	3	0
Adjuvant control group	Mean	201.773 n	186.485 n	186.715 n	524.570 n	431.325 n	478.120	128.770 n
	SD	13.438	131.105	105.211	139.974	115.679	76.710	.
	N	4	2	4	5	4	3	1
	%Diff	160.3	-12.2	70.3	27.7	-10.4	10.8	.
0.5mL-dose group	Mean	329.910 n	. n	231.472 n	627.557 n	350.510 n	662.836 *	136.960 n
	SD	.	.	83.674	193.623	.	134.197	.
	N	1	0	5	3	1	5	1
	%Diff	325.6	.	111.1	52.8	-27.2	53.7	.
1.5mL-dose group	Mean	77.510 n	. n	238.278 n	397.087 n	400.940 n	703.848 *	. n
	SD	.	.	57.005	30.659	99.193	60.448	.
	N	1	0	5	3	2	5	0
	%Diff	0.0	.	117.3	-3.3	-16.7	63.2	.

[I] - n - Inappropriate for statistics; [g] - ANOVA & Dunnett: * = p ≤ 0.05

Sex: F		Cytokines				
		TNF-α [I]	TNF-α [I]	IFN-γ [I]	IFN-γ [I]	IFN-γ [I]
		D60	D85	D4	D60	D85
Normal saline control group	Mean	132.860 n	. n	. n	. n	108.610 n
	SD	.	.	.	.	.
	N	1	0	0	0	1
Adjuvant control group	Mean	. n	. n	257.788 n	142.650 n	278.550 n
	SD	.	.	22.728	.	92.817
	N	0	0	5	1	5
	%Diff	.	.	.	.	156.5
0.5mL-dose group	Mean	. n	190.655 n	254.005 n	125.690 n	402.616 n
	SD	.	46.818	181.465	.	141.647
	N	0	2	2	1	5
	%Diff	.	.	.	.	270.7
1.5mL-dose group	Mean	. n	154.817 n	. n	. n	350.326 n
	SD	.	17.199	.	.	90.880
	N	0	3	0	0	5
	%Diff	.	.	.	.	222.6

[I] - n - Inappropriate for statistics

Table S3. Statistical data of organ weights (D60).

Sex: M		Organ weights						
		Brain (g) [g]	Thymus (g) [g]	Heart (g) [g]	Lung with bronchus (g) [g]	Liver (g) [g]	Spleen (g) [g]	Kidney (g) [g]
Normal saline control group	Mean	2.1311	0.3363	1.5644	1.7230	12.1486	0.7787	3.0520
	SD	0.0790	0.0861	0.1267	0.0970	0.9865	0.1184	0.3152
	N	10	10	10	10	10	10	10
Adjuvant control group	Mean	2.1510	0.4129	1.6588	1.8161	14.0525*	0.8548	3.2967
	SD	0.0863	0.0928	0.2067	0.2314	1.8299	0.1119	0.4418
	N	10	10	10	10	10	10	10
	%Diff	0.9	22.8	6.0	5.4	15.7	9.8	8.0
0.5mL-dose group	Mean	2.0478	0.3992	1.5683	1.7029	12.7052	0.8767	3.0325
	SD	0.1031	0.0810	0.1688	0.1886	1.3036	0.1188	0.3035
	N	10	10	10	10	10	10	10
	%Diff	-3.9	18.7	0.2	-1.2	4.6	12.6	-0.6
1.5mL-dose group	Mean	2.0989	0.3830	1.5932	1.7367	12.1434	0.9452*	2.9201
	SD	0.0498	0.1030	0.1688	0.1523	0.8962	0.1518	0.2849
	N	10	10	10	10	10	10	10
	%Diff	-1.5	13.9	1.8	0.8	0.0	21.4	-4.3

Sex: M		Organ weights						
		Adrenal gland (g) [g]	Testis (g) [g]	Epididymis (g) [g]	Prostate gland (g) [g]	Thyroid gland and parathyroid glands (g) [g]	Pituitary gland (g) [g1]	Seminal vesicle (g) [g]
Normal saline control group	Mean	0.0553	3.3559	1.2385	1.4038	0.0288	0.0106	1.5852
	SD	0.0077	0.4101	0.1238	0.1689	0.0043	0.0035	0.2411
	N	10	10	10	10	10	10	10
Adjuvant control group	Mean	0.0552	3.4023	1.3228	1.4335	0.0277	0.0121	1.5661
	SD	0.0092	0.4128	0.1517	0.2849	0.0054	0.0020	0.2693
	N	10	10	10	10	10	10	10
	%Diff	-0.2	1.4	6.8	2.1	-3.8	14.2	-1.2
0.5mL-dose group	Mean	0.0551	3.4221	1.2971	1.5142	0.0285	0.0112	1.6314
	SD	0.0098	0.3498	0.1357	0.2250	0.0051	0.0015	0.2541
	N	10	10	10	10	10	10	10
	%Diff	-0.4	2.0	4.7	7.9	-1.0	5.7	2.9
1.5mL-dose group	Mean	0.0555	3.3926	1.2903	1.3595	0.0276	0.0113	1.5604
	SD	0.0097	0.2954	0.1140	0.1893	0.0071	0.0007	0.1906
	N	10	10	10	10	10	10	10
	%Diff	0.4	1.1	4.2	-3.2	-4.2	6.6	-1.6

Sex: F		Organ weights						
		Brain (g) [g]	Thymus (g) [g]	Heart (g) [g]	Lung with bronchus (g) [g]	Liver (g) [g]	Spleen (g) [g]	Kidney (g) [g1]
Normal saline control group	Mean	1.9761	0.3405	1.0197	1.2699	7.6105	0.6188	1.8284
	SD	0.0787	0.0493	0.0988	0.0916	1.1384	0.2035	0.2174
	N	10	10	10	10	10	10	10
Adjuvant control group	Mean	1.9560	0.3081	1.0066	1.2806	8.0270	0.5425	1.8561
	SD	0.0792	0.0709	0.1437	0.1166	1.2593	0.0856	0.1802
	N	10	10	10	10	10	10	10
	%Diff	-1.0	-9.5	-1.3	0.8	5.5	-12.3	1.5
0.5mL-dose group	Mean	1.8644*	0.2847	0.9686	1.2710	7.6231	0.6362	1.8475
	SD	0.1149	0.0588	0.0404	0.1020	0.9410	0.0893	0.1145
	N	10	10	10	10	10	10	10
	%Diff	-5.7	-16.4	-5.0	0.1	0.2	2.8	1.0

1.5mL-dose group	Mean	1.9122	0.2869	0.9715	1.3421	8.2394	0.6965	1.9265
	SD	0.0701	0.0398	0.0825	0.1138	0.7701	0.0828	0.1198
	N	10	10	10	10	10	10	10
	%Diff	-3.2	-15.7	-4.7	5.7	8.3	12.6	5.4

**Sex: F**		**Organ weights**				
		**Adrenal gland (g) [g]**	**Uterus with cervix (g) [g1]**	**Ovary with fallopian tube (g) [g1]**	**Thyroid gland and parathyroid glands (g) [g]**	**Pituitary gland (g) [g]**
Normal saline control group	Mean	0.0609	0.6155	0.1337	0.0246	0.0157
	SD	0.0096	0.1860	0.0215	0.0032	0.0035
	N	10	10	10	10	10
Adjuvant control group	Mean	0.0653	0.5654	0.1495	0.0256	0.0166
	SD	0.0114	0.1928	0.0094	0.0042	0.0022
	N	10	10	10	10	10
	%Diff	7.2	-8.1	11.8	4.1	5.7
0.5mL-dose group	Mean	0.0628	0.6207	0.1469	0.0251	0.0163
	SD	0.0098	0.1385	0.0302	0.0043	0.0030
	N	10	10	10	10	10
	%Diff	3.1	0.8	9.9	2.0	3.8
1.5mL-dose group	Mean	0.0657	0.8295	0.1341	0.0232	0.0168
	SD	0.0112	0.4429	0.0249	0.0052	0.0027
	N	10	10	10	10	10
	%Diff	7.9	34.8	0.3	-5.7	7.0

(g) ANOVA&Dunnett *=p≤0.05
[g1] Kruskal Wallis &Wilcoxon *=p≤0.05

Table S4. Statistical data of blood cell count.

Sex: M		Blood Cell Count					
		WBC (10^9/L) [g]	WBC (10^9/L) [g]	WBC (10^9/L) [g]	Neut (10^9/L) [g1]	Neut (10^9/L) [g1]	Neut (10^9/L) [g]
		D4	D60	D85	D4	D60	D85
Normal saline control group	Mean	12.478	9.533	10.008	1.884	1.322	1.546
	SD	3.663	2.330	1.844	0.612	0.616	0.562
	N	5	10	5	5	10	5
Adjuvant control group	Mean	13.732	9.442	11.164	2.770	1.987 *	1.606
	SD	3.212	1.992	2.791	1.807	0.825	0.467
	N	5	10	5	5	10	5
	%Diff	10.0	-1.0	11.6	47.0	50.3	3.9
0.5mL-dose group	Mean	12.404	11.523	9.488	1.934	3.097 *	1.096
	SD	1.939	2.476	3.101	0.370	0.695	0.333
	N	5	10	5	5	10	5
	%Diff	-0.6	20.9	-5.2	2.7	134.3	-29.1
1.5mL-dose group	Mean	11.520	13.564 *	9.424	2.242	4.718 *	1.220
	SD	1.134	3.465	2.470	0.809	1.492	0.322
	N	5	10	5	5	10	5
	%Diff	-7.7	42.3	-5.8	19.0	256.9	-21.1

[g] - ANOVA & Dunnett: * = p ≤ 0.05; [g1] - ANOVA & Dunnett (Log): * = p ≤ 0.05

Sex: M		Blood Cell Count					
		Neut (%) [g]	Neut (%) [g1]	Neut (%) [g1]	Lymph (10^9/L) [g1]	Lymph (10^9/L) [g1]	Lymph (10^9/L) [g1]
		D4	D60	D85	D4	D60	D85
Normal saline control group	Mean	15.14	13.72	15.28	9.942	7.842	8.058
	SD	3.22	3.79	3.90	3.025	1.869	1.445
	N	5	10	5	5	10	5

Adjuvant control group	Mean	19.64	21.13 *	14.42	10.172	6.987	8.996
	SD	10.41	7.98	2.60	2.584	1.709	2.298
	N	5	10	5	5	10	5
	%Diff	29.7	54.0	-5.6	2.3	-10.9	11.6
0.5mL-dose group	Mean	15.84	27.72 *	11.72	9.790	7.819	7.928
	SD	3.78	6.87	2.25	1.801	2.240	2.665
	N	5	10	5	5	10	5
	%Diff	4.6	102.0	-23.3	-1.5	-0.3	-1.6
1.5mL-dose group	Mean	19.20	34.46 *	13.60	8.640	8.047	7.822
	SD	5.48	4.74	4.51	0.721	1.920	2.450
	N	5	10	5	5	10	5
	%Diff	26.8	151.2	-11.0	-13.1	2.6	-2.9

[g] - Kruskal-Wallis & Wilcoxon; [g1] - ANOVA & Dunnett: * = $p \leqslant 0.05$

Sex: M		Blood Cell Count					
		Lymph (%) [g]	Lymph (%) [g1]	Lymph (%) [g1]	Mono (10^9/L) [g1]	Mono (10^9/L) [g2]	Mono (10^9/L) [g1]
		D4	D60	D85	D4	D60	D85
Normal saline control group	Mean	79.56	82.24	80.66	0.306	0.145	0.192
	SD	2.78	4.28	3.91	0.083	0.044	0.051
	N	5	10	5	5	10	5
Adjuvant control group	Mean	74.56	73.94 *	80.48	0.466	0.252 *	0.268
	SD	10.99	7.87	2.50	0.124	0.053	0.078
	N	5	10	5	5	10	5
	%Diff	-6.3	-10.1	-0.2	52.3	73.8	39.6
0.5mL-dose group	Mean	78.66	67.06 *	83.36	0.388	0.308 *	0.238
	SD	4.11	6.95	2.49	0.064	0.092	0.117
	N	5	10	5	5	10	5
	%Diff	-1.1	-18.5	3.3	26.8	112.4	24.0
1.5mL-dose group	Mean	75.24	59.81 *	82.16	0.348	0.424 *	0.194
	SD	5.48	4.71	5.16	0.072	0.151	0.034
	N	5	10	5	5	10	5
	%Diff	-5.4	-27.3	1.9	13.7	192.4	1.0

[g] - Kruskal-Wallis & Wilcoxon; [g1] - ANOVA & Dunnett: * = $p \leqslant 0.05$; [g2] - ANOVA & Dunnett (Log): * = $p \leqslant 0.05$

Sex: M		Blood Cell Count					
		Mono (%) [g]	Mono (%) [g]	Mono (%) [g]	Eos (10^9/L) [g]	Eos (10^9/L) [g]	Eos (10^9/L) [g]
		D4	D60	D85	D4	D60	D85
Normal saline control group	Mean	2.52	1.56	1.90	0.108	0.116	0.130
	SD	0.56	0.50	0.40	0.049	0.056	0.020
	N	5	10	5	5	10	5
Adjuvant control group	Mean	3.38	2.71 *	2.46	0.126	0.116	0.154
	SD	0.48	0.58	0.77	0.077	0.043	0.057
	N	5	10	5	5	10	5
	%Diff	34.1	73.7	29.5	16.7	0.0	18.5
0.5mL-dose group	Mean	3.12	2.66 *	2.48	0.098	0.165	0.148
	SD	0.40	0.51	0.61	0.020	0.068	0.029
	N	5	10	5	5	10	5
	%Diff	23.8	70.5	30.5	-9.3	42.2	13.8
1.5mL-dose group	Mean	3.06	3.07 *	2.18	0.100	0.174	0.104
	SD	0.63	0.78	0.66	0.023	0.079	0.040
	N	5	10	5	5	10	5
	%Diff	21.4	96.8	14.7	-7.4	50.0	-20.0

[g] - ANOVA & Dunnett: * = $p \leqslant 0.05$

Sex：M		Blood Cell Count					
		Eos (%) [g]	Eos (%) [g]	Eos (%) [g]	Baso (10^9/L) [g]	Baso (10^9/L) [g]	Baso (10^9/L) [g]
		D4	D60	D85	D4	D60	D85
Normal saline control group	Mean	0.92	1.32	1.30	0.038	0.026	0.016
	SD	0.53	0.84	0.28	0.019	0.011	0.009
	N	5	10	5	5	10	5
Adjuvant control group	Mean	0.98	1.22	1.46	0.054	0.021	0.026
	SD	0.64	0.33	0.63	0.025	0.009	0.015
	N	5	10	5	5	10	5
	%Diff	6.5	-7.6	12.3	42.1	-19.2	62.5
0.5mL-dose group	Mean	0.80	1.47	1.64	0.042	0.025	0.018
	SD	0.19	0.49	0.53	0.018	0.008	0.013
	N	5	10	5	5	10	5
	%Diff	-13.0	11.4	26.2	10.5	-3.8	12.5
1.5mL-dose group	Mean	0.86	1.26	1.12	0.038	0.032	0.024
	SD	0.26	0.55	0.39	0.008	0.013	0.009
	N	5	10	5	5	10	5
	%Diff	-6.5	-4.5	-13.8	0.0	23.1	50.0

[g] - ANOVA & Dunnett

Sex：M		Blood Cell Count					
		Baso (%) [g]	Baso (%) [g]	Baso (%) [g]	RBC (10^12/L) [g1]	RBC (10^12/L) [g]	RBC (10^12/L) [g]
		D4	D60	D85	D4	D60	D85
Normal saline control group	Mean	0.32	0.26	0.18	6.576	9.027	9.058
	SD	0.04	0.08	0.08	0.266	0.329	0.319
	N	5	10	5	5	10	5
Adjuvant control group	Mean	0.36	0.23	0.24	6.570	8.894	9.272
	SD	0.11	0.05	0.05	0.391	0.291	0.417
	N	5	10	5	5	10	5
	%Diff	12.5	-11.5	33.3	-0.1	-1.5	2.4
0.5mL-dose group	Mean	0.34	0.23	0.18	6.664	8.394	9.270
	SD	0.11	0.07	0.08	0.170	1.284	0.321
	N	5	10	5	5	10	5
	%Diff	6.3	-11.5	0.0	1.3	-7.0	2.3
1.5mL-dose group	Mean	0.34	0.23	0.24	6.816	8.829	9.384
	SD	0.05	0.05	0.05	0.436	0.394	0.534
	N	5	10	5	5	10	5
	%Diff	6.3	-11.5	33.3	3.6	-2.2	3.6

[g] - ANOVA & Dunnett; [g1] - Kruskal-Wallis & Wilcoxon

Sex：M		Blood Cell Count					
		HGB (g/L) [g]	HGB (g/L) [g1]	HGB (g/L) [g]	HCT (%) [g]	HCT (%) [g1]	HCT (%) [g]
		D4	D60	D85	D4	D60	D85
Normal saline control group	Mean<br>SD<br>N	139.4<br>5.1<br>5	156.9<br>3.8<br>10	155.4<br>4.6<br>5	42.68<br>0.89<br>5	50.56<br>0.78<br>10	49.78<br>1.47<br>5
Adjuvant control group	Mean<br>SD<br>N<br>%Diff	137.4<br>7.7<br>5<br>-1.4	153.7<br>3.2<br>10<br>-2.0	156.4<br>5.3<br>5<br>0.6	42.68<br>2.44<br>5<br>0.0	49.42 *<br>1.26<br>10<br>-2.3	50.64<br>1.80<br>5<br>1.7
0.5mL-dose group	Mean<br>SD<br>N<br>%Diff	137.6<br>3.6<br>5<br>-1.3	145.1 *<br>19.6<br>10<br>-7.5	159.4<br>3.4<br>5<br>2.6	42.34<br>1.07<br>5<br>-0.8	47.07 *<br>6.54<br>10<br>-6.9	51.34<br>1.03<br>5<br>3.1
1.5mL-dose group	Mean<br>SD<br>N<br>%Diff	139.4<br>3.6<br>5<br>0.0	146.0 * 4.6<br>10<br>-6.9	157.4<br>5.6<br>5<br>1.3	43.30<br>1.84<br>5<br>1.5	47.74 *<br>1.65<br>10<br>-5.6	50.58<br>2.14<br>5<br>1.6

[g] - ANOVA & Dunnett; [g1] - Kruskal-Wallis & Wilcoxon: * = p ≤ 0.05

Sex：M		Blood Cell Count					
		Retic (10^12/L) [g]	Retic (10^12/L) [g]	Retic (10^12/L) [g]	Retic (%) [g]	Retic (%) [g]	Retic (%) [g]
		D4	D60	D85	D4	D60	D85
Normal saline control group	Mean<br>SD<br>N	0.3392<br>0.0304<br>5	0.2019<br>0.0331<br>10	0.1940<br>0.0187<br>5	5.154<br>0.289<br>5	2.239<br>0.350<br>10	2.142<br>0.218<br>5
Adjuvant control group	Mean<br>SD<br>N<br>%Diff	0.3508<br>0.0436<br>5<br>3.4	0.2097<br>0.0276<br>10<br>3.9	0.1992<br>0.0289<br>5<br>2.7	5.366<br>0.804<br>5<br>4.1	2.367<br>0.377<br>10<br>5.7	2.154<br>0.332<br>5<br>0.6
0.5mL-dose group	Mean<br>SD<br>N<br>%Diff	0.2624 *<br>0.0405<br>5<br>-22.6	0.2211<br>0.0346<br>10<br>9.5	0.1680<br>0.0495<br>5<br>-13.4	3.936 *<br>0.600<br>5<br>-23.6	2.672<br>0.446<br>10<br>19.3	1.822<br>0.576<br>5<br>-14.9
1.5mL-dose group	Mean<br>SD<br>N<br>%Diff	0.1834 *<br>0.0235<br>5<br>-45.9	0.1907<br>0.0306<br>10<br>-5.5	0.1978<br>0.0470<br>5<br>2.0	2.714 *<br>0.477<br>5<br>-47.3	2.172<br>0.401<br>10<br>-3.0	2.132<br>0.613<br>5<br>-0.5

[g] - ANOVA & Dunnett: * = p ≤ 0.05

Sex: M		Blood Cell Count					
		MCV (fL) [g]	MCV (fL) [g1]	MCV (fL) [g1]	MCHC (g/L) [g1]	MCHC (g/L) [g1]	MCHC (g/L) [g]
		D4	D60	D85	D4	D60	D85
Normal saline control group	Mean	64.96	56.06	55.00	326.6	310.3	312.2
	SD	1.54	1.66	2.22	7.2	6.6	2.0
	N	5	10	5	5	10	5
Adjuvant control group	Mean	64.98	55.59	54.64	322.0	311.1	309.2
	SD	1.18	2.06	1.42	6.0	6.6	4.8
	N	5	10	5	5	10	5
	%Diff	0.0	-0.8	-0.7	-1.4	0.3	-1.0
0.5mL-dose group	Mean	63.56	56.30	55.42	325.2	308.6	310.2
	SD	0.35	2.25	1.70	3.3	5.8	0.8
	N	5	10	5	5	10	5
	%Diff	-2.2	0.4	0.8	-0.4	-0.5	-0.6
1.5mL-dose group	Mean	63.64	54.14	54.04	322.4	305.9	311.4
	SD	1.82	2.69	3.00	8.6	6.5	3.6
	N	5	10	5	5	10	5
	%Diff	-2.0	-3.4	-1.7	-1.3	-1.4	-0.3

[g] - Kruskal-Wallis & Wilcoxon; [g1] - ANOVA & Dunnett

Sex: M		Blood Cell Count					
		MCH (pg) [g]	MCH (pg) [g1]	MCH (pg) [g1]	PLT (10^9/L) [g]	PLT (10^9/L) [g1]	PLT (10^9/L) [g1]
		D4	D60	D85	D4	D60	D85
Normal saline control group	Mean	21.22	17.39	17.18	1135.6	961.9	925.6
	SD	0.63	0.65	0.66	88.8	70.9	116.6
	N	5	10	5	5	10	5
Adjuvant control group	Mean	20.92	17.30	16.90	1096.6	969.6	982.6
	SD	0.34	0.63	0.51	399.5	134.6	85.9
	N	5	10	5	5	10	5
	%Diff	-1.4	-0.5	-1.6	-3.4	0.8	6.2
0.5mL-dose group	Mean	20.66	17.36	17.20	1258.4	974.9	891.8
	SD	0.26	0.80	0.55	179.5	183.5	94.6
	N	5	10	5	5	10	5
	%Diff	-2.6	-0.2	0.1	10.8	1.4	-3.7
1.5mL-dose group	Mean	20.50	16.54 *	16.82	1242.8	1054.8	904.2
	SD	1.07	0.53	0.79	31.3	102.5	81.1
	N	5	10	5	5	10	5
	%Diff	-3.4	-4.9	-2.1	9.4	9.7	-2.3

[g] - Kruskal-Wallis & Wilcoxon [g1] - ANOVA & Dunnett: * = $p \leqslant 0.05$

Sex: F		Blood Cell Count					
		WBC (10^9/L) [g]	WBC (10^9/L) [g]	WBC (10^9/L) [g1]	Neut (10^9/L) [g]	Neut (10^9/L) [g2]	Neut (10^9/L) [g]
		D4	D60	D85	D4	D60	D85
Normal saline control group	Mean	11.308	7.002	5.984	0.782	0.834	0.762
	SD	3.920	2.339	1.740	0.166	0.441	0.186
	N	5	10	5	5	10	5
Adjuvant control group	Mean	12.102	6.750	6.922	1.252	0.833	1.060
	SD	1.990	1.714	1.485	0.460	0.339	0.677
	N	5	10	5	5	10	5
	%Diff	7.0	-3.6	15.7	60.1	-0.1	39.1
0.5mL-dose group	Mean	11.870	9.532	8.178	1.286	2.448 *	0.784
	SD	3.042	2.266	3.228	0.573	0.958	0.392
	N	5	10	5	5	10	5
	%Diff	5.0	36.1	36.7	64.5	193.5	2.9
1.5mL-dose group	Mean	10.542	10.973 *	7.292	1.406	2.752 *	0.578
	SD	2.566	3.121	0.618	0.242	0.957	0.112
	N	5	10	5	5	10	5
	%Diff	-6.8	56.7	21.9	79.8	230.0	-24.1

[g] - ANOVA & Dunnett: * = p ≤ 0.05; [g1] - Kruskal-Wallis & Wilcoxon; [g2] - ANOVA & Dunnett (Log): * = p ≤ 0.05

Sex: F		Blood Cell Count					
		Neut (%) [g]	Neut (%) [g]	Neut (%) [g1]	Lymph (10^9/L) [g1]	Lymph (10^9/L) [g1]	Lymph (10^9/L) [g2]
		D4	D60	D85	D4	D60	D85
Normal saline control group	Mean	7.40	11.52	13.56	10.058	5.871	4.964
	SD	2.30	3.02	4.49	3.760	1.875	1.695
	N	5	10	5	5	10	5
Adjuvant control group	Mean	10.22	12.28	15.64	10.236	5.535	5.520
	SD	2.83	3.27	9.77	1.571	1.398	1.595
	N	5	10	5	5	10	5
	%Diff	38.1	6.6	15.3	1.8	-5.7	11.2
0.5mL-dose group	Mean	11.30	24.87 *	9.48	10.054	6.591	6.996
	SD	4.65	6.48	2.12	2.928	1.419	2.743
	N	5	10	5	5	10	5
	%Diff	52.7	115.9	-30.1	0.0	12.3	40.9
1.5mL-dose group	Mean	13.56	25.36 *	8.08	8.520	7.580	6.376
	SD	1.41	6.82	2.15	2.152	2.428	0.683
	N	5	10	5	5	10	5
	%Diff	83.2	120.1	-40.4	-15.3	29.1	28.4

[g] - ANOVA & Dunnett(Log): * = p ≤ 0.05; [g1] - ANOVA & Dunnett; [g2] - Kruskal-Wallis & Wilcoxon

Sex: F		Blood Cell Count					
		Lymph (%) [g]	Lymph (%) [g1]	Lymph (%) [g1]	Mono (10^9/L) [g1]	Mono (10^9/L) [g1]	Mono (10^9/L) [g1]
		D4	D60	D85	D4	D60	D85
Normal saline control group	Mean<br>SD<br>N	88.42<br>2.47<br>5	84.19<br>3.56<br>10	82.02<br>4.72<br>5	0.202<br>0.044<br>5	0.149<br>0.084<br>10	0.120<br>0.025<br>5
Adjuvant control group	Mean<br>SD<br>N<br>%Diff	84.82<br>3.77<br>5<br>-4.1	82.02<br>3.95<br>10<br>-2.6	79.38<br>9.95<br>5<br>-3.2	0.278<br>0.123<br>5<br>37.6	0.197<br>0.054<br>10<br>32.2	0.154<br>0.042<br>5<br>28.3
0.5mL-dose group	Mean<br>SD<br>N<br>%Diff	84.16<br>5.48<br>5<br>-4.8	69.93 *<br>7.24<br>10<br>-16.9	85.60<br>1.97<br>5<br>4.4	0.236<br>0.124<br>5<br>16.8	0.228<br>0.075<br>10<br>53.0	0.170<br>0.061<br>5<br>41.7
1.5mL-dose group	Mean<br>SD<br>N<br>%Diff	80.68 *<br>1.34<br>5<br>-8.8	68.91 *<br>6.72<br>10<br>-18.1	87.30<br>2.23<br>5<br>6.4	0.296<br>0.145<br>5<br>46.5	0.343 *<br>0.129<br>10<br>130.2	0.144<br>0.038<br>5<br>20.0

[g] - Kruskal-Wallis & Wilcoxon: * = p ≤ 0.05 [g1] - ANOVA & Dunnett: * = p ≤ 0.05

Sex: F		Blood Cell Count					
		Mono (%) [g]	Mono (%) [g]	Mono (%) [g]	Eos (10^9/L) [g]	Eos (10^9/L) [g]	Eos (10^9/L) [g]
		D4	D60	D85	D4	D60	D85
Normal saline control group	Mean<br>SD<br>N	1.84<br>0.38<br>5	2.11<br>0.71<br>10	2.06<br>0.43<br>5	0.108<br>0.028<br>5	0.064<br>0.020<br>10	0.084<br>0.017<br>5
Adjuvant control group	Mean<br>SD<br>N<br>%Diff	2.24<br>0.86<br>5<br>21.7	3.02 *<br>0.76<br>10<br>43.1	2.26<br>0.61<br>5<br>9.7	0.136<br>0.059<br>5<br>25.9	0.081<br>0.031<br>10<br>26.6	0.114<br>0.055<br>5<br>35.7
0.5mL-dose group	Mean<br>SD<br>N<br>%Diff	1.98<br>0.87<br>5<br>7.6	2.37<br>0.63<br>10<br>12.3	2.10<br>0.25<br>5<br>1.9	0.126<br>0.026<br>5<br>16.7	0.160 *<br>0.063<br>10<br>150.0	0.108<br>0.027<br>5<br>28.6
1.5mL-dose group	Mean<br>SD<br>N<br>%Diff	2.70<br>0.72<br>5<br>46.7	3.07 *<br>0.45<br>10<br>45.5	1.94<br>0.40<br>5<br>-5.8	0.154<br>0.097<br>5<br>42.6	0.151 *<br>0.104<br>10<br>135.9	0.096<br>0.024<br>5<br>14.3

[g] - ANOVA & Dunnett: * = p ≤ 0.05

Sex: F		Blood Cell Count					
		Eos (%) [g]	Eos (%) [g]	Eos (%) [g1]	Baso (10^9/L) [g1]	Baso (10^9/L) [g]	Baso (10^9/L) [g1]
		D4	D60	D85	D4	D60	D85
Normal saline control group	Mean<br>SD<br>N	0.98<br>0.25<br>5	0.95<br>0.28<br>10	1.46<br>0.46<br>5	0.050<br>0.037<br>5	0.017<br>0.008<br>10	0.008<br>0.004<br>5
Adjuvant control group	Mean<br>SD<br>N<br>%Diff	1.10<br>0.36<br>5<br>12.2	1.22<br>0.36<br>10<br>28.4	1.68<br>0.74<br>5<br>15.1	0.048<br>0.018<br>5<br>-4.0	0.010<br>0.005<br>10<br>-41.2	0.010<br>0.007<br>5<br>25.0
0.5mL-dose group	Mean<br>SD<br>N<br>%Diff	1.08<br>0.08<br>5<br>10.2	1.73 *<br>0.69<br>10<br>82.1	1.40<br>0.30<br>5<br>-4.1	0.052<br>0.026<br>5<br>4.0	0.019<br>0.009<br>10<br>11.8	0.020<br>0.010<br>5<br>150.0
1.5mL-dose group	Mean<br>SD<br>N<br>%Diff	1.60<br>1.41<br>5<br>63.3	1.30<br>0.48<br>10<br>36.8	1.30<br>0.23<br>5<br>-11.0	0.032<br>0.022<br>5<br>-36.0	0.026<br>0.016<br>10<br>52.9	0.012<br>0.004<br>5<br>50.0

[g] - ANOVA & Dunnett (Log): * = p ≤ 0.05; [g1] - ANOVA & Dunnett

Sex: F		Blood Cell Count					
		Baso (%) [g]	Baso (%) [g]	Baso (%) [g]	RBC (10^12/L) [g]	RBC (10^12/L) [g]	RBC (10^12/L) [g]
		D4	D60	D85	D4	D60	D85
Normal saline control group	Mean<br>SD<br>N	0.40<br>0.16<br>5	0.24<br>0.05<br>10	0.14<br>0.09<br>5	7.368<br>0.261<br>5	7.939<br>0.398<br>10	7.896<br>0.097<br>5
Adjuvant control group	Mean<br>SD<br>N<br>%Diff	0.38<br>0.13<br>5<br>-5.0	0.16<br>0.05<br>10<br>-33.3	0.14<br>0.09<br>5<br>0.0	7.632<br>0.193<br>5<br>3.6	8.015<br>0.437<br>10<br>1.0	8.054<br>0.379<br>5<br>2.0
0.5mL-dose group	Mean<br>SD<br>N<br>%Diff	0.42<br>0.11<br>5<br>5.0	0.21<br>0.06<br>10<br>-12.5	0.24<br>0.05<br>5<br>71.4	7.678<br>0.456<br>5<br>4.2	7.932<br>0.446<br>10<br>-0.1	8.410<br>0.255<br>5<br>6.5
1.5mL-dose group	Mean<br>SD<br>N<br>%Diff	0.28<br>0.13<br>5<br>-30.0	0.21<br>0.09<br>10<br>-12.5	0.18<br>0.08<br>5<br>28.6	7.352<br>0.170<br>5<br>-0.2	7.933<br>0.273<br>10<br>-0.1	8.148<br>0.270<br>5<br>3.2

[g] - ANOVA & Dunnett

Sex: F		Blood Cell Count					
		HGB (g/L) [g]	HGB (g/L) [g]	HGB (g/L) [g]	HCT (%) [g]	HCT (%) [g]	HCT (%) [g]
		D4	D60	D85	D4	D60	D85
Normal saline control group	Mean	150.0	146.4	146.4	43.94	46.72	46.24
	SD	5.1	7.7	3.5	1.55	2.28	1.37
	N	5	10	5	5	10	5
Adjuvant control group	Mean	152.2	144.6	150.6	45.04	46.51	47.90
	SD	1.5	7.9	4.6	0.56	3.07	1.92
	N	5	10	5	5	10	5
	%Diff	1.5	-1.2	2.9	2.5	-0.4	3.6
0.5mL-dose group	Mean	153.6	143.4	150.4	46.12	45.66	47.98
	SD	4.8	6.1	2.5	2.18	2.09	1.53
	N	5	10	5	5	10	5
	%Diff	2.4	-2.0	2.7	5.0	-2.3	3.8
1.5mL-dose group	Mean	150.0	140.3	148.4	44.24	45.86	47.88
	SD	4.4	6.1	2.5	1.30	1.83	0.85
	N	5	10	5	5	10	5
	%Diff	0.0	-4.2	1.4	0.7	-1.8	3.5

[g] - ANOVA & Dunnet

Sex: F		Blood Cell Count					
		Retic (10^12/L) [g]	Retic (10^12/L) [g]	Retic (10^12/L) [g]	Retic (%) [g]	Retic (%) [g]	Retic (%) [g]
		D4	D60	D85	D4	D60	D85
Normal saline control group	Mean	0.1854	0.1806	0.1926	2.516	2.294	2.442
	SD	0.0342	0.0348	0.0248	0.451	0.514	0.320
	N	5	10	5	5	10	5
Adjuvant control group	Mean	0.1586	0.1728	0.1604	2.074	2.175	1.998
	SD	0.0299	0.0304	0.0328	0.354	0.469	0.463
	N	5	10	5	5	10	5
	%Diff	-14.5	-4.3	-16.7	-17.6	-5.2	-18.2
0.5mL-dose group	Mean	0.1418 *	0.1802	0.1386	1.850 *	2.285	1.652
	SD	0.0160	0.0299	0.0271	0.254	0.440	0.364
	N	5	10	5	5	10	5
	%Diff	-23.5	-0.2	-28.0	-26.5	-0.4	-32.4
1.5mL-dose group	Mean	0.1230 *	0.1824	0.1800	1.676 *	2.303	2.210
	SD	0.0142	0.0259	0.0486	0.187	0.359	0.580
	N	5	10	5	5	10	5
	%Diff	-33.7	1.0	-6.5	-33.4	0.4	-9.5

[g] - ANOVA & Dunnett: * = $p \leqslant 0.05$

Sex: F		Blood Cell Count					
		MCV (fL) [g]	MCV (fL) [g]	MCV (fL) [g]	MCHC (g/L) [g]	MCHC (g/L) [g]	MCHC (g/L) [g]
		D4	D60	D85	D4	D60	D85
Normal saline control group	Mean	59.64	58.86	58.58	341.0	313.0	316.8
	SD	1.21	1.33	1.85	2.0	7.1	6.5
	N	5	10	5	5	10	5
Adjuvant control group	Mean	59.04	58.00	59.48	338.0	311.0	314.2
	SD	0.89	1.44	2.11	2.9	6.9	8.8
	N	5	10	5	5	10	5
	%Diff	-1.0	-1.5	1.5	-0.9	-0.6	-0.8
0.5mL-dose group	Mean	60.14	57.65	57.08	333.2	314.2	313.6
	SD	2.17	2.49	2.26	7.0	5.6	5.7
	N	5	10	5	5	10	5
	%Diff	0.8	-2.1	-2.6	-2.3	0.4	-1.0
1.5mL-dose group	Mean	60.18	57.82	58.84	339.0	306.3	310.4
	SD	0.63	1.80	1.94	5.5	6.1	5.9
	N	5	10	5	5	10	5
	%Diff	0.9	-1.8	0.4	-0.6	-2.1	-2.0

[g] - ANOVA & Dunnett

Sex: F		Blood Cell Count					
		MCH (pg) [g]	MCH (pg) [g1]	MCH (pg) [g]	PLT (10^9/L) [g]	PLT (10^9/L) [g]	PLT (10^9/L) [g]
		D4	D60	D85	D4	D60	D85
Normal saline control group	Mean	20.36	18.43	18.56	1112.0	1008.5	886.0
	SD	0.51	0.27	0.45	193.7	117.3	49.2
	N	5	10	5	5	10	5
Adjuvant control group	Mean	19.96	18.05	18.70	1075.8	975.2	967.6
	SD	0.43	0.35	0.58	97.8	94.0	87.8
	N	5	10	5	5	10	5
	%Diff	-2.0	-2.1	0.8	-3.3	-3.3	9.2
0.5mL-dose group	Mean	20.04	18.10	17.88	1166.2	1037.0	894.8
	SD	0.75	0.75	0.48	149.3	89.4	87.4
	N	5	10	5	5	10	5
	%Diff	-1.6	-1.8	-3.7	4.9	2.8	1.0
1.5mL-dose group	Mean	20.38	17.71	18.24	1162.0	1139.2 *	912.8
	SD	0.29	0.72	0.79	241.4	93.0	102.1
	N	5	10	5	5	10	5
	%Diff	0.1	-3.9	-1.7	4.5	13.0	3.0

[g] - ANOVA & Dunnett: * = $p \leqslant 0.05$ [g1] - Kruskal-Wallis & Wilcoxon

Table S5. Statistical data of coagulation function.

Sex: M		PT (Seconds) [g]	PT (Seconds) [g1]	APTT (Seconds) [g1]	APTT (Seconds) [g1]	FIB (g/L) [g1]	FIB (g/L) [g1]
		Coagulation Function					
		D60	D85	D60	D85	D2	D4
Normal saline control group	Mean	8.92	9.34	13.98	13.50	2.9142	3.0718
	SD	0.19	0.13	1.19	0.84	0.1744	0.2646
	N	10	5	10	5	5	5
Adjuvant control group	Mean	8.91	9.36	12.71 *	14.24	3.3190	3.0256
	SD	0.17	0.22	1.12	1.69	0.2608	0.2176
	N	10	5	10	5	5	5
	%Diff	-0.1	0.2	-9.1	5.5	13.9	-1.5
0.5mL-dose group	Mean	8.86	9.42	12.63 *	14.72	5.0220 *	3.4512 *
	SD	0.36	0.28	1.07	0.79	0.5299	0.2003
	N	10	5	10	5	5	5
	%Diff	-0.7	0.9	-9.7	9.0	72.3	12.4
1.5mL-dose group	Mean	8.68	9.36	12.16 *	13.78	6.8958 *	4.1004 *
	SD	0.15	0.22	1.02	1.17	0.3099	0.2367
	N	10	5	10	5	5	5
	%Diff	-2.7	0.2	-13.0	2.1	136.6	33.5

[g] - Kruskal-Wallis & Wilcoxon [g1] - ANOVA & Dunnett: * = p ≤ 0.05

Sex: M		FIB (g/L) [g]	FIB (g/L) [g]	FIB (g/L) [g]
		Coagulation Function		
		D58	D60	D85
Normal saline control group	Mean	2.7672	2.5703	2.6088
	SD	0.1644	0.2794	0.0972
	N	5	10	5
Adjuvant control group	Mean	3.2852 *	3.0064 *	2.6428
	SD	0.3000	0.1888	0.1717
	N	5	10	5
	%Diff	18.7	17.0	1.3
0.5mL-dose group	Mean	3.7082 *	3.2916 *	2.6804
	SD	0.3321	0.3325	0.1325
	N	5	10	5
	%Diff	34.0	28.1	2.7
1.5mL-dose group	Mean	4.4990 *	3.8673 *	2.4692
	SD	0.1821	0.3912	0.1805
	N	5	10	5
	%Diff	62.6	50.5	-5.4

[g] - ANOVA & Dunnett: * = p ≤ 0.05

Sex: F		Coagulation Function					
		PT (Seconds) [g]	PT (Seconds) [g]	APTT (Seconds) [g1]	APTT (Seconds) [g]	FIB (g/L) [g]	FIB (g/L) [g]
		D60	D85	D60	D85	D2	D4
Normal saline control group	Mean	8.05	8.42	11.28	12.38	2.5968	2.3208
	SD	0.17	0.23	1.13	0.59	0.1698	0.1270
	N	10	5	10	5	5	5
Adjuvant control group	Mean	8.05	8.32	12.21	12.06	3.0584	2.7420
	SD	0.15	0.28	1.31	1.04	0.4289	0.6112
	N	10	5	10	5	5	5
	%Diff	0.0	-1.2	8.2	-2.6	17.8	18.1
0.5mL-dose group	Mean	8.02	8.40	11.66	12.08	4.7920 *	3.2366 *
	SD	0.18	0.29	0.80	1.47	0.5716	0.4161
	N	10	5	10	5	5	5
	%Diff	-0.4	-0.2	3.4	-2.4	84.5	39.5
1.5mL-dose group	Mean	7.92	8.50	10.98	13.40	6.5228 *	3.4914 *
	SD	0.14	0.22	0.37	1.65	0.7328	0.3265
	N	10	5	10	5	5	5
	%Diff	-1.6	1.0	-2.7	8.2	151.2	50.4

[g] - ANOVA & Dunnett: * = p ≤ 0.05; [g1] - Kruskal-Wallis & Wilcoxon

Sex: F		Coagulation Function		
		FIB (g/L) [g]	FIB (g/L) [g]	FIB (g/L) [g]
		D58	D60	D85
Normal saline control group	Mean	2.5650	2.2413	1.8130
	SD	0.5247	0.5805	0.1648
	N	5	10	5
Adjuvant control group	Mean	2.8706	2.1198	1.8162
	SD	0.3266	0.2102	0.1805
	N	5	10	5
	%Diff	11.9	-5.4	0.2
0.5mL-dose group	Mean	3.0628	2.5469	1.8198
	SD	0.6453	0.3870	0.0504
	N	5	10	5
	%Diff	19.4	13.6	0.4
1.5mL-dose group	Mean	3.9482 *	2.7260 *	1.6722
	SD	0.4066	0.1983	0.0994
	N	5	10	5
	%Diff	53.9	21.6	-7.8

[g] - ANOVA & Dunnett: * = p ≤ 0.05

Table S6. Statistical data of blood biochemistry.

Sex: M		Blood biochemistry					
		ALT (U/L) [g]	ALT (U/L) [g]	AST (U/L) [g]	AST (U/L) [g]	ALP (U/L) [g1]	ALP (U/L) [g]
		D60	D85	D60	D85	D60	D85
Normal saline control group	Mean	40.3	33.8	116.0	98.4	138.5	114.0
	SD	4.8	2.9	25.7	17.5	28.1	12.1
	N	10	5	10	5	10	5
Adjuvant control group	Mean	40.2	40.2	110.3	148.2	129.3	119.2
	SD	11.4	1.9	15.2	18.2	18.4	20.9
	N	10	5	10	5	10	5
	%Diff	-0.2	18.9	-4.9	50.6	-6.6	4.6
0.5mL-dose group	Mean	41.7	41.4 *	114.2	145.4	112.3 *	119.0
	SD	10.9	6.7	29.9	58.4	13.4	22.5
	N	10	5	10	5	10	5
	%Diff	3.5	22.5	-1.6	47.8	-18.9	4.4
1.5mL-dose group	Mean	35.0	35.8	121.3	98.6	102.2 *	113.0
	SD	5.1	3.0	25.7	25.2	12.2	31.0
	N	10	5	10	5	10	5
	%Diff	-13.2	5.9	4.6	0.2	-26.2	-0.9

[g] - ANOVA & Dunnett: * = p ≤ 0.05; [g1] - ANOVA & Dunnett (Log): * = p ≤ 0.05

Sex: M		Blood biochemistry					
		TBil (μmol/L) [g]	TBil (μmol/L) [g]	GGT (U/L) [g1]	GGT (U/L) [g]	TP (g/L) [g]	TP (g/L) [g]
		D60	D85	D60	D85	D60	D85
Normal saline control group	Mean	2.838	3.064	0.1	0.8	63.47	68.46
	SD	0.294	0.337	0.3	0.4	1.94	3.13
	N	10	5	10	5	10	5
Adjuvant control group	Mean	2.766	2.840	0.0	0.6	64.37	69.20
	SD	0.346	0.272	0.0	0.5	2.21	2.73
	N	10	5	10	5	10	5
	%Diff	-2.5	-7.3	-100.0	-25.0	1.4	1.1
0.5mL-dose group	Mean	2.720	3.000	0.0	0.4	66.00 *	71.86
	SD	0.315	0.182	0.0	0.5	1.71	2.09
	N	10	5	10	5	10	5
	%Diff	-4.2	-2.1	-100.0	-50.0	4.0	5.0
1.5mL-dose group	Mean	2.845	2.970	0.1	0.6	65.28	72.42 *
	SD	0.261	0.267	0.3	0.5	2.00	1.30
	N	10	5	10	5	10	5
	%Diff	0.2	-3.1	0.0	-25.0	2.9	5.8

[g] - ANOVA & Dunnett: * = p ≤ 0.05 [g1] - Kruskal-Wallis & Wilcoxon

Sex: M		Blood biochemistry					
		Alb (g/L) [g]	Alb (g/L) [g1]	A/G [g]	A/G [g]	Glu (mmol/L) [g]	Glu (mmol/L) [g]
		D60	D85	D60	D85	D60	D85
Normal saline control group	Mean	32.90	34.00	1.09	0.98	7.184	7.210
	SD	1.15	1.92	0.09	0.04	0.717	0.739
	N	10	5	10	5	10	5
Adjuvant control group	Mean	32.04	34.12	0.99 *	0.96	7.090	6.756
	SD	1.25	0.52	0.06	0.05	0.831	0.719
	N	10	5	10	5	10	5
	%Diff	-2.6	0.4	-9.2	-2.0	-1.3	-6.3
0.5mL-dose group	Mean	30.51 *	34.70	0.88 *	0.94	6.916	7.052
	SD	0.77	1.19	0.06	0.05	0.453	0.638
	N	10	5	10	5	10	5
	%Diff	-7.3	2.1	-19.3	-4.1	-3.7	-2.2
1.5mL-dose group	Mean	28.02 *	34.30	0.74 *	0.90	6.756	7.496
	SD	0.74	1.00	0.05	0.07	0.544	0.851
	N	10	5	10	5	10	5
	%Diff	-14.8	0.9	-32.1	-8.2	-6.0	4.0

[g] - ANOVA & Dunnett: * = p ≤ 0.05[g1] - Kruskal-Wallis & Wilcoxon

Sex: M		Blood biochemistry					
		UREA (mmol/L) [g]	UREA (mmol/L) [g]	Cre (μmol/L) [g]	Cre (μmol/L) [g]	CK (U/L) [g]	CK (U/L) [g]
		D60	D85	D60	D85	D60	D85
Normal saline control group	Mean	5.48	5.14	28.8	32.2	377.8	229.6
	SD	0.82	0.85	2.8	3.6	184.2	66.8
	N	10	5	10	5	10	5
Adjuvant control group	Mean	5.47	5.04	29.0	34.4	269.7	362.8
	SD	0.75	0.69	2.2	2.7	103.7	200.0
	N	10	5	10	5	10	5
	%Diff	-0.2	-1.9	0.7	6.8	-28.6	58.0
0.5mL-dose group	Mean	5.46	4.98	29.4	32.8	250.5	206.4
	SD	0.42	0.39	1.8	1.6	159.8	51.0
	N	10	5	10	5	10	5
	%Diff	-0.4	-3.1	2.1	1.9	-33.7	-10.1
1.5mL-dose group	Mean	5.60	5.38	30.6	33.4	307.9	297.2
	SD	0.62	0.96	2.7	3.4	192.2	248.9
	N	10	5	10	5	10	5
	%Diff	2.2	4.7	6.3	3.7	-18.5	29.4

[g] - ANOVA & Dunnett

Sex: M		Blood biochemistry					
		LDH (U/L) [g]	LDH (U/L) [g]	CHO (mmol/L) [g]	CHO (mmol/L) [g1]	TG (mmol/L) [g2]	TG (mmol/L) [g]
		D60	D85	D60	D85	D60	D85
Normal saline control group	Mean	1055.4	769.8	1.648	2.208	0.775	1.112
	SD	543.3	244.9	0.278	0.657	0.240	0.298
	N	10	5	10	5	10	5
Adjuvant control group	Mean	730.6	902.0	2.064 *	1.606	0.788	0.804
	SD	327.7	519.8	0.393	0.171	0.189	0.275
	N	10	5	10	5	10	5
	%Diff	-30.8	17.2	25.2	-27.3	1.7	-27.7
0.5mL-dose group	Mean	671.4	513.2	1.719	1.868	0.651	0.996
	SD	462.9	88.2	0.352	0.275	0.168	0.237
	N	10	5	10	5	10	5
	%Diff	-36.4	-33.3	4.3	-15.4	-16.0	-10.4
1.5mL-dose group	Mean	843.3	774.4	1.324	1.972	0.552 *	0.838
	SD	633.6	644.6	0.181	0.633	0.100	0.172
	N	10	5	10	5	10	5
	%Diff	-20.1	0.6	-19.7	-10.7	-28.8	-24.6

[g] - ANOVA & Dunnett: * = p ≤ 0.05; [g1] - Kruskal-Wallis & Wilcoxon; [g2] - ANOVA & Dunnett(Log): * = p ≤ 0.05

Sex: M		Blood biochemistry					
		Ca (mmol/L) [g]	Ca (mmol/L) [g1]	P (mmol/L) [g]	P (mmol/L) [g]	Na+ (mmol/L) [g]	Na+ (mmol/L) [g]
		D60	D85	D60	D85	D60	D85
Normal saline control group	Mean	2.687	2.704	2.454	2.236	142.28	142.90
	SD	0.081	0.140	0.142	0.285	1.44	1.06
	N	10	5	10	5	10	5
Adjuvant control group	Mean	2.721	2.664	2.407	2.318	142.11	142.42
	SD	0.113	0.062	0.141	0.061	1.02	0.97
	N	10	5	10	5	10	5
	%Diff	1.3	-1.5	-1.9	3.7	-0.1	-0.3
0.5mL-dose group	Mean	2.693	2.734	2.445	2.168	142.77	142.70
	SD	0.068	0.061	0.213	0.158	1.31	0.51
	N	10	5	10	5	10	5
	%Diff	0.2	1.1	-0.4	-3.0	0.3	-0.1
1.5mL-dose group	Mean	2.662	2.738	2.436	2.286	141.88	142.40
	SD	0.092	0.070	0.110	0.159	0.97	1.27
	N	10	5	10	5	10	5
	%Diff	-0.9	1.3	-0.7	2.2	-0.3	-0.3

[g] - ANOVA & Dunnett; [g1] - Kruskal-Wallis & Wilcoxon

Sex: M		Blood biochemistry			
		K^+ (mmol/L) [g]	K^+ (mmol/L) [g]	Cl^- (mmol/L) [g]	Cl^- (mmol/L) [g]
		D60	D85	D60	D85
Normal saline control group	Mean<br>SD<br>N	4.683<br>0.176<br>10	4.716<br>0.092<br>5	103.28<br>1.50<br>10	103.58<br>1.34<br>5
Adjuvant control group	Mean<br>SD<br>N<br>%Diff	4.647<br>0.239<br>10<br>-0.8	4.710<br>0.202<br>5<br>-0.1	102.65<br>1.00<br>10<br>-0.6	103.92<br>1.14<br>5<br>0.3
0.5mL-dose group	Mean<br>SD<br>N<br>%Diff	4.627<br>0.277<br>10<br>-1.2	4.626<br>0.131<br>5<br>-1.9	103.13<br>1.60<br>10<br>-0.1	103.68<br>1.72<br>5<br>0.1
1.5mL-dose group	Mean<br>SD<br>N<br>%Diff	4.777<br>0.359<br>10<br>2.0	4.652<br>0.226<br>5<br>-1.4	102.79<br>1.46<br>10<br>-0.5	103.66<br>1.25<br>5<br>0.1

[g] - ANOVA & Dunnet

Sex: F		Blood biochemistry					
		ALT (U/L) [g]	ALT (U/L) [g]	AST (U/L) [g1]	AST (U/L) [g2]	ALP (U/L) [g]	ALP (U/L) [g]
		D60	D85	D60	D85	D60	D85
Normal saline control group	Mean<br>SD<br>N	34.3<br>12.7<br>10	30.6<br>5.7<br>5	96.6<br>16.6<br>10	98.8<br>5.6<br>5	54.0<br>7.1<br>10	53.2<br>10.8<br>5
Adjuvant control group	Mean<br>SD<br>N<br>%Diff	32.4<br>8.4<br>10<br>-5.5	39.8<br>9.2<br>5<br>30.1	101.4<br>17.4<br>10<br>5.0	130.6<br>17.7<br>5<br>32.2	49.7<br>11.3<br>10<br>-8.0	50.2<br>13.6<br>5<br>-5.6
0.5mL-dose group	Mean<br>SD<br>N<br>%Diff	31.7<br>6.8<br>10<br>-7.6	40.4<br>9.2<br>5<br>32.0	103.0<br>12.5<br>10<br>6.6	116.6<br>29.8<br>5<br>18.0	50.6<br>14.6<br>10<br>-6.3	57.8<br>9.0<br>5<br>8.6
1.5mL-dose group	Mean<br>SD<br>N<br>%Diff	33.3<br>6.8<br>10<br>-2.9	38.4<br>6.6<br>5<br>25.5	130.5 *<br>26.4<br>10<br>35.1	93.0<br>13.8<br>5<br>-5.9	56.1<br>11.9<br>10<br>3.9	62.6<br>22.2<br>5<br>17.7

[g] - ANOVA & Dunnett; [g1] - ANOVA & Dunnett (Log): * = $p \leqslant 0.05$; [g2] - Kruskal-Wallis & Wilcoxon

Sex: F		Blood biochemistry					
		TBil (μmol/L) [g]	TBil (μmol/L) [g1]	GGT (U/L) [g1]	GGT (U/L) [g1]	TP (g/L) [g]	TP (g/L) [g1]
		D60	D85	D60	D85	D60	D85
Normal saline control group	Mean	3.496	3.872	0.0	0.6	72.82	80.20
	SD	0.540	0.402	0.0	0.5	3.72	6.22
	N	10	5	10	5	10	5
Adjuvant control group	Mean	3.331	3.694	0.2	1.0	72.13	80.10
	SD	0.352	0.225	0.4	0.0	4.75	3.38
	N	10	5	10	5	10	5
	%Diff	-4.7	-4.6	.	66.7	-0.9	-0.1
0.5mL-dose group	Mean	3.070	3.908	0.2	1.0	70.60	76.08
	SD	0.563	0.513	0.4	0.0	3.89	2.09
	N	10	5	10	5	10	5
	%Diff	-12.2	0.9	.	66.7	-3.0	-5.1
1.5mL-dose group	Mean	3.450	3.884	0.8	1.0	70.01	82.82
	SD	0.304	0.205	2.2	0.0	3.39	3.59
	N	10	5	10	5	10	5
	%Diff	-1.3	0.3	.	66.7	-3.9	3.3

[g] - ANOVA & Dunnett; [g1] - Kruskal-Wallis & Wilcoxon

Sex: F		Blood biochemistry					
		Alb (g/L) [g]	Alb (g/L) [g1]	A/G [g]	A/G [g]	Glu (mmol/L) [g]	Glu (mmol/L) [g]
		D60	D85	D60	D85	D60	D85
Normal saline control group	Mean	38.93	43.18	1.15	1.18	6.301	7.160
	SD	2.37	3.63	0.07	0.04	0.721	0.586
	N	10	5	10	5	10	5
Adjuvant control group	Mean	38.58	44.02	1.14	1.22	6.593	6.998
	SD	2.71	1.35	0.05	0.04	0.435	0.432
	N	10	5	10	5	10	5
	%Diff	-0.9	1.9	-0.9	3.4	4.6	-2.3
0.5mL-dose group	Mean	34.40 *	39.36	0.94 *	1.08	6.555	6.740
	SD	3.02	1.32	0.07	0.08	1.015	0.375
	N	10	5	10	5	10	5
	%Diff	-11.6	-8.8	-18.3	-8.5	4.0	-5.9
1.5mL-dose group	Mean	32.61 *	43.06	0.88 *	1.12	6.320	6.840
	SD	1.47	2.52	0.04	0.08	0.775	0.519
	N	10	5	10	5	10	5
	%Diff	-16.2	-0.3	-23.5	-5.1	0.3	-4.5

[g] - ANOVA & Dunnett: * = $p \leqslant 0.05$; [g1] - ANOVA & Dunnett (Log)

Sex: F		Blood biochemistry					
		UREA (mmol/L) [g]	UREA (mmol/L) [g]	Cre (µmol/L) [g]	Cre (µmol/L) [g1]	CK (U/L) [g]	CK (U/L) [g]
		D60	D85	D60	D85	D60	D85
Normal saline control group	Mean	5.42	5.14	34.6	36.6	205.7	215.4
	SD	0.67	0.52	3.3	2.1	77.6	33.1
	N	10	5	10	5	10	5
Adjuvant control group	Mean	5.61	6.12	35.0	39.0	175.4	401.6
	SD	0.69	1.22	2.3	2.6	48.2	275.7
	N	10	5	10	5	10	5
	%Diff	3.5	19.1	1.2	6.6	-14.7	86.4
0.5mL-dose group	Mean	5.79	5.94	35.5	40.4	190.5	224.0
	SD	0.64	0.93	3.5	4.4	94.6	77.8
	N	10	5	10	5	10	5
	%Diff	6.8	15.6	2.6	10.4	-7.4	4.0
1.5mL-dose group	Mean	5.90	5.06	34.1	34.0	236.1	203.6
	SD	0.90	0.78	2.2	5.2	133.5	94.4
	N	10	5	10	5	10	5
	%Diff	8.9	-1.6	-1.4	-7.1	14.8	-5.5

[g] - ANOVA & Dunnett; [g1] - Kruskal-Wallis & Wilcoxon

Sex: F		Blood biochemistry					
		LDH (U/L) [g]	LDH (U/L) [g]	CHO (mmol/L) [g]	CHO (mmol/L) [g]	TG (mmol/L) [g]	TG (mmol/L) [g]
		D60	D85	D60	D85	D60	D85
Normal saline control group	Mean	537.8	700.0	2.236	2.438	0.556	0.648
	SD	171.8	162.2	0.521	0.499	0.245	0.187
	N	10	5	10	5	10	5
Adjuvant control group	Mean	480.7	1169.2	2.046	2.622	0.567	0.836
	SD	195.4	627.8	0.398	0.520	0.123	0.438
	N	10	5	10	5	10	5
	%Diff	-10.6	67.0	-8.5	7.5	2.0	29.0
0.5mL-dose group	Mean	554.7	683.8	1.790	2.476	0.457	0.672
	SD	295.3	253.5	0.470	0.411	0.117	0.117
	N	10	5	10	5	10	5
	%Diff	3.1	-2.3	-19.9	1.6	-17.8	3.7
1.5mL-dose group	Mean	632.7	583.8	1.863	2.244	0.549	0.618
	SD	423.5	357.9	0.321	0.444	0.225	0.289
	N	10	5	10	5	10	5
	%Diff	17.6	-16.6	-16.7	-8.0	-1.3	-4.6

[g] - ANOVA & Dunnett

Sex: F		Blood biochemistry					
		Ca (mmol/L) [g]	Ca (mmol/L) [g1]	P (mmol/L) [g2]	P (mmol/L) [g2]	Na+ (mmol/L) [g2]	Na+ (mmol/L) [g2]
		D60	D85	D60	D85	D60	D85
Normal saline control group	Mean	2.844	2.860	2.092	1.758	141.52	141.52
	SD	0.121	0.111	0.267	0.250	1.10	0.77
	N	10	5	10	5	10	5
Adjuvant control group	Mean	2.839	2.886	2.095	1.722	140.71	141.18
	SD	0.142	0.104	0.330	0.182	1.49	1.17
	N	10	5	10	5	10	5
	%Diff	-0.2	0.9	0.1	-2.0	-0.6	-0.2
0.5mL-dose group	Mean	2.738	2.806	1.944	1.838	139.92	140.26
	SD	0.147	0.049	0.340	0.181	1.09	1.33
	N	10	5	10	5	10	5
	%Diff	-3.7	-1.9	-7.1	4.6	-1.1	-0.9
1.5mL-dose group	Mean	2.749	2.886	2.131	1.826	140.54	141.26
	SD	0.055	0.151	0.184	0.173	1.26	1.38
	N	10	5	10	5	10	5
	%Diff	-3.3	0.9	1.9	3.9	-0.7	-0.2

[g] - ANOVA & Dunnett (Log); [g1] - Kruskal-Wallis & Wilcoxon; [g2] - ANOVA & Dunnett

Sex: F		Blood biochemistry			
		K^+ (mmol/L) [g]	K^+ (mmol/L) [g]	Cl^- (mmol/L) [g]	Cl^- (mmol/L) [g]
		D60	D85	D60	D85
Normal saline control group	Mean	4.283	3.994	103.04	104.22
	SD	0.426	0.226	2.12	1.22
	N	10	5	10	5
Adjuvant control group	Mean	4.234	4.074	103.82	103.24
	SD	0.214	0.239	1.69	0.69
	N	10	5	10	5
	%Diff	-1.1	2.0	0.8	-0.9
0.5mL-dose group	Mean	4.199	4.194	104.24	104.04
	SD	0.332	0.173	1.88	1.39
	N	10	5	10	5
	%Diff	-2.0	5.0	1.2	-0.2
1.5mL-dose group	Mean	4.492	4.104	104.39	105.30
	SD	0.212	0.212	1.32	1.38
	N	10	5	10	5
	%Diff	4.9	2.8	1.3	1.0

[g] - ANOVA & Dunnet

Table S7. The tissues that were examined by light microscopy.

Tissue/Organ	Microscopy	Tissue/Organ	Microscopy	Tissue/Organ	Microscopy
Site of drug administration	Granulomatous inflammation	Large Intestine (Cecum, Colon, Rectum)	Normal	Seminal Vesicle	Normal
Adrenal gland	Normal	Larynx	Normal	Skeletal Muscle (Biceps Femoris)	Normal
Aorta	Normal	Liver	Normal	Skin (Around the Mammary Gland)	Normal
Bone and bone marrow, femur and sternum	Normal	Lung and Bronchus	Normal	Small Intestine (Duodenum, Jejunum, Ileum)	Normal
Brain	Normal	Lymph Nodes (Mandibular Lymph Node, Mesenteric Lymph	Normal	Spinal Cord (Cervical, Thoracic, Lumbar)	Normal

		Node, Inguinal Lymph Node)			
Epididymis	Normal	Mammary Gland	Normal	Spleen	Normal
Esophagus	Normal	Sciatic Nerve	Normal	Stomach (Glandular Stomach and Nonglandular Stomach)	Normal
Eye and optic nerve	Normal	Ovary and Oviduct	Normal	Testis	Normal
Harderian gland	Normal	Peyer's Patch	Normal	Thymus	Normal
Heart	Normal	Pancreas	Normal	Thyroid and Parathyroid Glands	Normal
Kidney	Normal	Prostate	Normal	Tongue	Normal
Lacrimal Gland	Normal	Pituitary Gland	Normal	Trachea	Normal
Uterus and Cervix	Normal	Salivary Glands, 3 Pairs	Normal	Urinary Bladder	Normal
Vagina	Normal				
